# A Multi-Point Contact Model Considering Rough Surface for Linear Ultrasonic Motors: Validation and Simulation

**DOI:** 10.3390/mi13111988

**Published:** 2022-11-16

**Authors:** Ying He, Zhiyuan Yao, Hao Xu

**Affiliations:** State Key Laboratory of Mechanics and Control of Mechanical Structures, Nanjing University of Aeronautics and Astronautics, Nanjing 210016, China

**Keywords:** ultrasonic motor, contact analysis, measurement, simulation, rough surface

## Abstract

The performance and wear life of linear ultrasonic motors are directly determined by the stator–mover frictional contact behaviors. A complete contact model is important to clearly understand the stator–mover contact mechanism and accurately estimate the motor performance. In this paper, a multi-point frictional contact model considering the roughness of contact interfaces is presented based on a finite model of the stator and an analytical model of the mover. The static/dynamic contact behaviors and output performance of the motor can be simulated efficiently. A quantitative measuring methodology for the dynamic contact forces between the stator and mover is developed. The effectiveness of the contact model for simulating the stator–mover contact forces is first evaluated by experiment. Based on the developed model, several dynamic characteristics of a linear ultrasonic motor are discussed: (a) the static force transferred between contact interfaces under pre-pressure; (b) the transient forces and energy exchange between contact interfaces; (c) the steady-state output performance of motor under different electric excitation parameters; (d) the effects of micro-topography parameters on the output performance of the motor and the force transmission of the contact interface.

## 1. Introduction

A linear ultrasonic motor (LUM), which can convert the ultrasonic-frequency oscillation of a special piezoelectric actuator into macroscopic linear motion directly by means of frictional contact, has many advantages, such as fast step/settle, unlimited travel ranges, self-locking at rest, and satisfying the compatibility of the magnetic field and vacuum. These advantages are a perfect match for many demands in precision motion control applications including precision stages [1], aerospace [2], medical devices [3,4,5], and semiconductor manufacturing [6]. It is obvious that the frictional contact behaviors between the stator and mover in an LUM not only determines the operational performance but also the wear properties and lifetime. Therefore, many researchers tried to understand the frictional contact mechanism. However, this subject is still a current challenge in both theoretical and experimental studies.

In theories, many kinds of frictional contact models for LUM have been proposed to describe the interaction of the interface between the stator and mover. Tsai et al. [7] presented a single point contact model for a so-called L1B2 ultrasonic motor, in which the normal contact force was equivalent to a linear elastic spring force and the tangential contact force was expressed by Coulomb model. The dynamic force transmission at contact interfaces and some output characteristics of the motor were simulated when the stator was excited by square wave voltage. Based on this model and considering the stick–slip-separation dynamic behavior of the contact interface, Li et al. [8] developed a switch model for the same motor exited by sine-wave voltage. Considering that the vibration amplitude of the stator and the roughness of the contact surface were in the same order of magnitude, Lv et al. [9] refined the normal contact model by taking the rough surface into account and obtained a more accurate estimation for the output performance. Deng et al. [10] proposed a parametric model for the speed control of a motor, in which some unknown physics parameters due to lack of prior accurate knowledge were considered and identified by experiment. However, the above frictional contact models were coupled with an analytical stator model for the simulation of contact mechanism and motor performance, which could cause invalidation or lead to inaccurate results when they are directly used for irregular-structured LUMs. Scholars have to make some assumptions and simplifications for investigating the frictional contact behavior in LUMs with complex structure stators. Shi et al. [11] presented a widely applied contact modeling method for standing wave linear ultrasonic motors with the assumption that the contact process does not affect the vibration characteristics of the stator. With a similar assumption, a modified Coulomb friction model was developed in [12,13], and the dynamic contact, friction drive, and wear mechanisms in an LUM with a V-shape stator was outlined. All the existing analytical models treated the stator–mover contact interface as point-to-point contact, which may not enable us to understand the frictional contact properties between the stator and mover in detail because the size of the contact interface is much larger than the vibration amplitude of the stator. In [14], a multi-point contact model was developed based on the finite element method (FEM); however, the effect of surface roughness on the frictional contact behavior was neglected. Although the FEM could model a randomly rough surface at the contact surface of the stator, this would result in a multi-scale stator model which would need a tremendous amount of calculation. Some other relevant studies that concentrate on the frictional contact of the linear ultrasonic motor can be found in [15,16,17,18,19]. Because of those, it is interesting to develop a detailed multi-point contact model considering the surface roughness of the stator–mover interface, which would be useful for investigating the contact mechanism in more detail and for accurately predicting the output performance of the motor.

In previous studies, the accuracy of a proposed stator–mover contact model was generally validated by comparing the output performance of the motor between simulation and experiment results. Within the knowledge of the author, the load information at the stator–mover contact interface has never been analyzed quantitatively by experiment, and only a few relevant studies are devoted to the qualitative measurement of the frictional contact forces at contact interfaces. In [20,21], an electric contact method is adopted for measuring the stator–mover contact state. A resistor, DC power, stator, and a conducting mover were connected in series, and then the contact and separation periods can be discriminated by measuring the voltage across the resistor. The influences of the exciting voltage amplitude and the pre-load pressing stator against the mover on the contact time in one running cycle of the stator were investigated. However, the information obtained from the electric contact method is not enough to analyze the interfacial contact forces quantitatively. In fact, two of the major obstacles to studying motor wear and developing a control strategy are the inability to quantitatively measure the interfacial contact forces and the lack of an accurate contact model verified by experiment. Therefore, an experimental method for measuring the normal contact force and frictional force at the contact interfaces of LUMs is necessary. Additionally, the reasonability and reliability of the theoretical contact model can also be verified.

To address these issues, a multi-point contact model considering interfacial micro-topography parameters is presented based on the finite element model of the stator and the analytical model of the mover, which could be used to simulate the frictional contact behaviors and the output performance of LUMs. The static and dynamic contact algorithms were designed and implemented in a Matlab environment based on the Newton–Raphason method and Runge–Kutta method, respectively. The static and dynamic responses of the stator, mover, and contact forces can be output. Furthermore, a quantitative measuring methodology of the dynamic contact forces between the stator and mover was proposed for validating the effectiveness of simulation results. By comparing the simulated and experimental results, the proposed model is reliable and effective in investigating the interfacial contact mechanism and predicting the output performance of the motor.

This paper is organized as follows. In Section 2, the configuration and operating principle of an LUM are presented briefly. In Section 3, the numerical model for simulating the contact behaviors of linear ultrasonic motors is developed, in which the finite element model of the stator, analytical mover model, and the multi-point frictional contact model considering interfacial roughness are included. The solution algorithms for static and dynamic models are designed. Subsequently, the measurement principle and system for stator–mover contact forces are presented in Section 4. The model validation and some significant phenomena are simulated and discussed in Section 5. Finally, the conclusions are drawn in Section 6.

## 2. The LUM Prototype and Operating Principle

**Figure 1:**
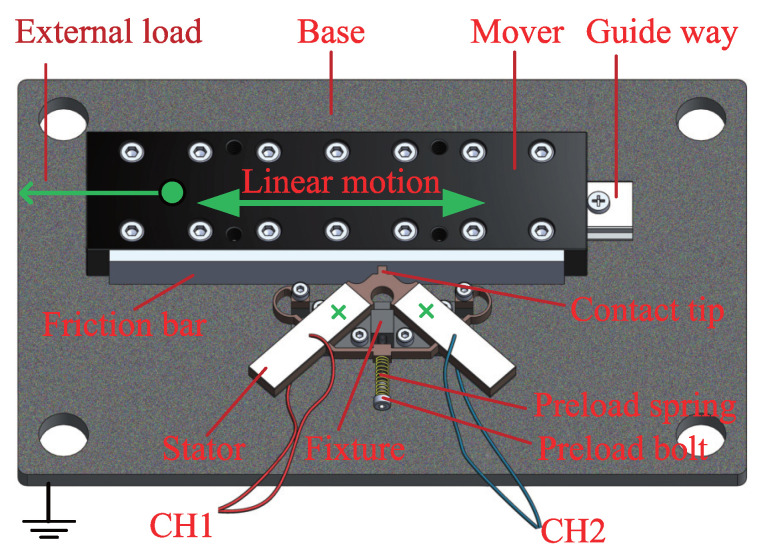
Structure of the LUM prototype.

**Figure 2:**
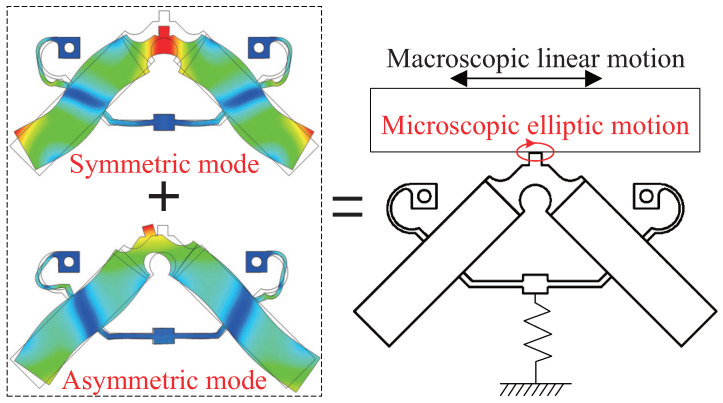
The operating principle description with the finite element model.

The configuration of the studied LUM is shown in Figure 1. The prototype motor consists of a V-shaped piezoelectric stator with a contact tip, a frame fixture clamping stator, a pre-pressing assemble including a preload spring and a preload bolt, a mover with a wear-resistant friction bar, and a base. Note that the external load may be applied to the mover. Four PZT-5H piezoelectric ceramics polarized in the thickness direction are symmetrically arranged onto the stator surface and electrically excited to induce particular resonant modes of the stator. When the surface electrodes on top (CH1/CH2) are applied sinusoidal signals with a specific frequency close to the resonant frequency and a phase difference, the symmetric and asymmetric modes ($f_{1}$ = 53,191 Hz and $f_{2}$ = 53,261 Hz, respectively) of the stator are simultaneously excited and the contact tip will generate a microscopic elliptic motion, as shown in Figure 2. Since the stator is preloaded against the mover, the mover will be driven to generate a macroscopic linear motion by the contact tip through frictional contact behaviors. The frictional contact process between the contact tip and mover directly determines the dynamic properties of the mover as well as the lifetime and reliability of the motor.

## 3. Numerical Modeling and Algorithm

In this section, the numerical model for simulating the stator–mover contact behavior and the output performance of LUM is developed, where the stator is modeled based on the finite element method, the mover is simplified as a single-degree-freedom system, and the stator–mover contact interface is modeled considering the interfacial roughness and the multi-point contact effect. The solution algorithms for the static and dynamic models are designed based on the Newton–Raphason method and the Runge–Kutta method, respectively.

### 3.1. Stator and Mover Model

**Figure 3:**
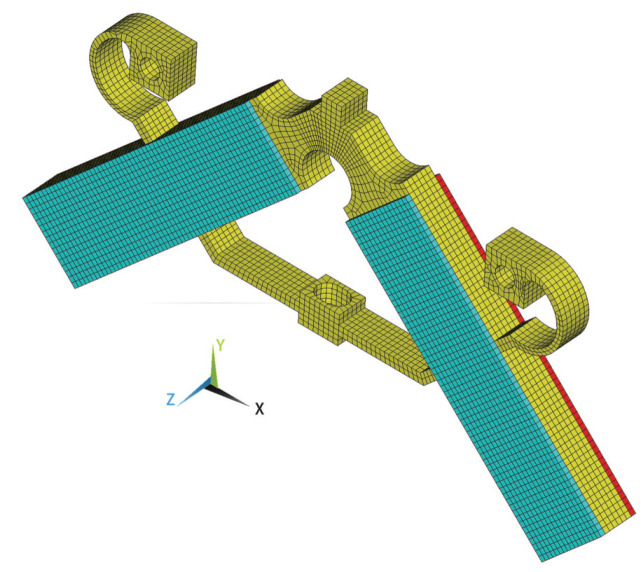
The 3D finite element mesh of the stator.

In order to build a multi-point contact model incorporating micro-topography parameters of the contact surface, a 3D stator including PZT and metal substrate is modeled by the finite method, as shown in Figure 3. Here, 25,735 nodes and 20,262 isoparametric cubic elements with 8 nodes are used, and the mesh size of 0.5 mm is taken for the contact interface. For static coupled-field analysis [22,23], the discretized equation of the stator can be expressed as

(1) $$\left[\begin{array}{cc} K_{uu} & K_{uV}\\ K_{Vu} & K_{VV} \end{array}\right]\left[\begin{array}{c} u\\ V \end{array}\right]=\left[\begin{array}{c} F\\ Q \end{array}\right],$$

where $K_{uu}$, $K_{uV}$, and $K_{VV}$ are the structure, coupling, conductivity stiffness matrix. The vectors u and V are the nodal displacement and electric potential, respectively. Q is the equivalent nodal electric charge, and it is usually set to zero because the electric excitation is applied through voltage. It is noted that the vector F is the equivalent force derived from the sum of all extended nodal forces including pre-pressure $F_{pre}$, normal contact force $F_{N}$, and tangential friction force $F_{T}$. The coupled finite element matrix equation for the dynamic analysis of the stator is given by

(2) $$\left[\begin{array}{cc} M_{uu} & 0\\ 0 & 0 \end{array}\right]\left[\begin{array}{c} \ddot{u}\\ \ddot{V} \end{array}\right]+C\left[\begin{array}{c} \dot{u}\\ \dot{V} \end{array}\right]+\left[\begin{array}{cc} K_{uu} & K_{uV}\\ K_{Vu} & K_{VV} \end{array}\right]\left[\begin{array}{c} u\\ V \end{array}\right]=\left[\begin{array}{c} F^{*}+F\\ Q \end{array}\right],$$

where C is the Rayleigh damping matrix, and the vector $F^{*}$ is generated from the electric excitation boundaries. The state of mover motion at any position is assumed to be the same and only moves along the guideway, so a single-degree-freedom system could be used to represent the mover, and the dynamic equation of the mover is easily obtained

(3) $$m{\ddot{u}}_{m}+c{\dot{u}}_{m}=F_{T_{m}}+F_{f}-F_{load},$$

where $u_{m}$, *m*, and *c* are the displacement, mass, and damping coefficient of the mover. Note that the damping coefficient of the mover only affects the maximum speed of the mover and the time reaching the speed steady-state, so it was determined by trial calculation and comparison with an experiment in one working condition. $F_{T_{m}}$, $F_{f}$, and $F_{load}$ are the driving force of all nodes at the contact tip, the friction force between the mover and guideway, and the external load applied to the mover.

By solving those coupling models, the frictional contact behavior and response characteristics in the LUM can be analyzed in detail. The key point for accurate simulation is to build a complete contact model. In addition, the strong nonlinearity of the frictional contact behavior leads to the solution of dynamic equations being significantly difficult and computationally time-consuming. An appropriate frictional contact model and solution algorithms are crucial for obtaining accurate solution results, which will be presented in the following sections.

### 3.2. Normal Contact Model Considering Rough Surface

The real stator–mover contact surfaces are composed of a series of rough peaks with random radii and heights. In the analysis of the normal contact problem, the shape of rough peaks is usually considered as a curved surface with the same radius of curvature *R*, and the interactions between roughness peaks are neglected. The height distribution of the roughness peaks is assumed to obey standard Gaussian distribution determined by the root mean square roughness. The contact problem between the contact tip surface and mover surface is equivalent to the contact between a rigid smooth surface and an elastic rough surface [24], as is shown in Appendix A.

Considering one asperity with the height $h^{\prime }$ and the penetration depth $\delta^{\prime }$, the normal contact force can be obtained based on the Hertz contact theory [25] and is expressed as

(4) $$F^{\prime }=\frac{4}{3}E^{*}R^{1/2}\delta^{\prime 3/2},$$

By taking a derivative of Equation (4) with respect to the penetration depth $\delta^{\prime }$, the variation of the normal contact stiffness with $\delta^{\prime }$ for an asperity is obtained as follows:

(5) $$k^{\prime }=2E^{*}\sqrt{R\delta^{\prime }},$$

If each asperity is regarded as a nonlinear spring, all of the asperities can be considered as parallel springs with random height, i.e., the normal contact stiffness of the rough surface is equal to the sum of stiffness of all asperities involved in contact. When the penetration depth of the rigid smooth surface is δ, the total normal contact stiffness can be obtained by integrating over all of the asperities with heights from $h=h_{max}-\delta$ to $h_{max}$:

(6) $$k=\int_{h}^{h_{max}}A\eta k^{\prime }\psi \left(h^{\prime }\right)d\left(h^{\prime }\right),$$

where *A* is the area of the contact tip surface. η is the areal asperity density determined by experiment [26]. Because the probability that the height of asperity lies in the range −3σ and 3σ is about 99.7%, the maximum peak height is selected as 3σ. Substituting Equation (5) into Equation (6), the total normal contact stiffness is given by:

(7) $$k\left(\delta \right)=2A\eta E^{*}\sqrt{\frac{R}{2\pi \sigma^{2}}}\int_{3\sigma -\delta }^{3\sigma }\sqrt{h^{\prime }-3\sigma +\delta }e^{-\frac{h^{\prime 2}}{2\sigma^{2}}}d\left(h^{\prime }\right).$$

By integrating Equation (7) and utilizing the numerical method in MATLAB, the total normal contact stiffness will be a function of the penetration depth of the rigid smooth surface.

The developments that follow here are to apply the normal contact model to the stator and mover models. For the discretized stator, the total normal contact force is assigned to each node on the contact tip so the normal contact stiffness for node *i* is calculated by

(8) $$k_{i}\left(\delta \right)=\frac{k(\delta )}{n},$$

where *n* is the total number of nodes on the contact tip. Then the normal contact force of this node with the penetration depth of $\delta_{i}$ is

(9) $$F_{N_{i}}\left(\delta_{i}\right)=-\int_{0}^{\delta_{i}}k_{i}\left(\delta \right)d\delta .$$

The penetration depth of $\delta_{i}$ is contributed by the pre-pressure and the electric excitation in the stator so that it is separated into two parts: the static penetration depth ${\tilde{u}}_{i}$ and the dynamic penetration depth $u_{i}$ at this contact node, which is written as

(10) $$\delta_{i}=\left\{\begin{array}{cc} {\tilde{u}}_{i}+u_{i} & {\tilde{u}}_{i}+u_{i}>0\\ 0 & {\tilde{u}}_{i}+u_{i}\leq 0 \end{array}\right.$$

Here, $\delta_{i}=0$ denotes the separation of this contact pair. All of the nodal normal contact forces are calculated and assembled into the global normal contact force vector $F_{N}$ of stator model. The normal contact force in the mover model is the counterforce of the resultant force of all nodal normal contact forces, which is expressed as

(11) $$F_{N_{m}}=-\sum_{i}^{n}F_{N_{i}}.$$

### 3.3. Regularization Friction Model

**Figure 4:**
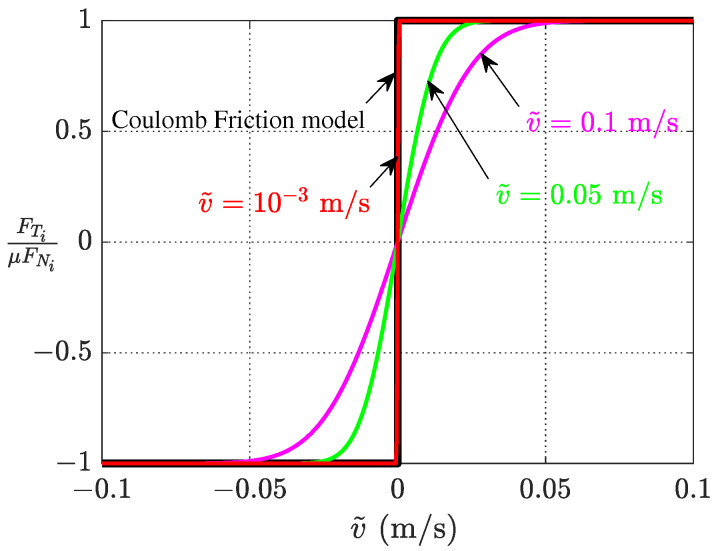
Approximation of actual friction force by a Regularization friction model.

In order to consider the stick condition in the stator–mover dynamic contact, a regularization friction model is developed to replace the discontinuous Coulomb friction laws which make the numerical procedures difficult due to the discontinuous jump of the relative tangential velocity in contact interface [27]. The frictional force of node *i* at the contact tip surface is expressed by the following continuous form:

(12) $$F_{{}_{T_{i}}}=\mu F_{{}_{N_{i}}}\mathrm{erf}\left(3.6\frac{v_{i}}{\tilde{v}}\right).$$

where μ is the friction coefficient of stator–mover contact pair, and erf denotes the Gauss error function. $v_{i}={\dot{u}}_{i}-{\dot{u}}_{m}$ is the relative velocity between the mover and this node, and $\tilde{v}$ is a stick–slip characteristic velocity differentiating between static and kinetic friction. In this form, the sticking behavior of the contact interface is treated as sliding at a velocity less than $\tilde{v}$ so that the frictional force in both the stick and sliding phases is expressed by the relation in Equation (11). Figure 4 shows the level of approximation between the Coulomb friction model and the regularization friction model with different stick–slip characteristic velocities of $\tilde{v}=0.1$ m/s, $\tilde{v}=0.05$ m/s, and $\tilde{v}=10^{-3}$ m/s. It can be seen that this model could agree well with the actual properties of the frictional force as long as the stick–slip characteristic velocity is small enough.

Similar to the normal contact model, this friction model is used for the stator and mover models. The global frictional force vector $F_{T}$ in the stator model and the driving force $F_{T_{m}}$ in the mover model can be obtained by all of the nodal frictional forces at contact tip surface. With this frictional contact model, the microscopic characteristics between the contact tip of the stator and the friction bar of the mover are taken into account. The whole model of the motor is obtained by combining the stator model, mover model, normal contact model, and friction model. The solution algorithm for the static/dynamic model will be presented in the following section.

### 3.4. Static Contact Algorithm

For the application of pre-pressure in LUM, the interfacial behavior between the contact tip and mover is considered static contact. Based on the static stator model and the normal contact model formulated in Equation (1), the stator model considering the static contact behavior can be rewritten as:

(13) $$G\left(a\right)=Ka-F_{pre}-F_{N}\left(a\right)=0$$

where $a={\left[\begin{array}{cc} u & V \end{array}\right]}^{T}$ is the unknown degree of freedom (DOF) values to be solved, and K is the global stiffness matrix of the coupling field. Equation (13) is a nonlinear equation because the normal contact forces vector $F_{N}\left(a\right)$ is a function of the unknown normal displacement on the contact tip. The general Newto-Raphason algorithm [28,29] is used to solve this nonlinear equation. The iteration format is constructed as

(14) $$a^{n+1}=a^{n}-{\left(\frac{\partial G}{\partial a}{|}_{a=a^{n}}\right)}^{-1}G\left(a^{n}\right)$$

where the superscript *n* is the number of iterations. $\frac{\partial G}{\partial a}$ is the system Jacobian matrix and given by

(15) $$\frac{\partial G}{\partial a}=K-k\left(a\right)$$

where $k\left(a\right)=\frac{\partial F_{N}}{\partial a}$ is called the contact stiffness matrix, and it is assembled from the contact stiffness of all nodes involved in contact which can be calculated in Equations (7) and (8). The contact stiffness of one node is placed at the corresponding *y*-DOF position.

**Figure 5:**
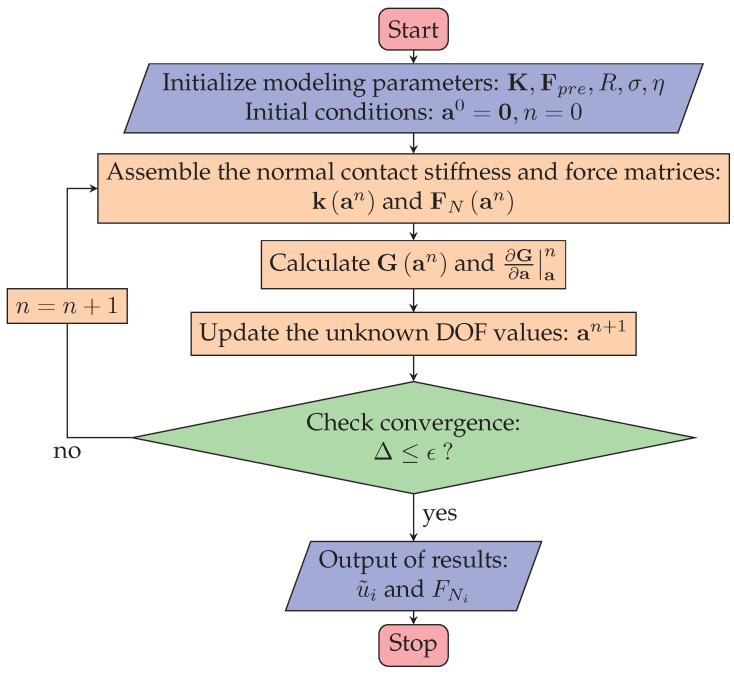
Flowchart of the static contact algorithm.

According to the normal contact model established in Section 3.2, Equation (13) is continuous and derivable when the nodal displacement on the surface of the contact tip is greater than or equal to 0. That is, this iterative algorithm converges when the initial nodal displacement on the contact surface is set to greater than or equal to zero. Therefore, the initial DOF values $a^{0}=0$ are used for the iterative format in Equation (14). The convergence condition is determined by the following equation:

(16) $$\Delta =∥a^{n+1}-a^{n}{∥}_{2}<\epsilon ,$$

where ϵ is the convergence tolerance. According to the above iteration format, an algorithm process for analyzing the stator–mover static contact behavior is designed, and the main steps are shown in Figure 5.

The static contact algorithm begins with the 3D stator model and the micro-topography parameters of the contact surface. The initial DOF values are considered to be zeros. When a pre-pressure is applied to the stator, the static penetration depth and contact forces can be calculated. The results of the calculation are not only a precondition of the dynamic contact algorithm, but can be used to simulate the actual pre-pressure obtained at the contact interface.

### 3.5. Dynamic Contact Algorithm

The dynamic contact problem of the LUM couples the dynamic stator model, the dynamic mover model, and the normal and tangential contact model, so its solution is complex and computationally time-consuming. The normalization of mode shapes is utilized to simplify the semi-discrete equation of stator motion given in Equation (2), where the mode shapes of the stator are assumed to be unaffected by the stator–mover contact behavior. Based on the frictional contact models presented in the preceding section the dynamic stator model is rewritten as

(17) $$M\ddot{a}\left(t\right)+C\dot{a}\left(t\right)+Ka\left(t\right)=F^{*}\left(t\right)+F_{pre}+F_{N}\left(\tilde{a},a\left(t\right)\right)+F_{T}\left(\tilde{a},a\left(t\right),\dot{a}\left(t\right),{\dot{u}}_{m}\left(t\right)\right),$$

where $\tilde{a}$ donates the static DOF values of the stator under pre-pressure, which is calculated utilizing the static contact algorithm.

Define a modal generalized displacements q(t) such that

**Figure 6:**
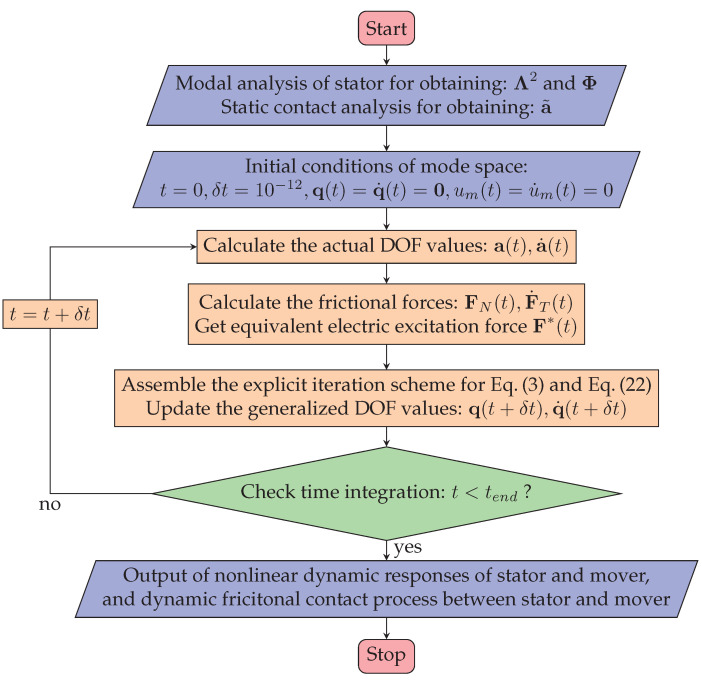
Flowchart of the dynamic contact algorithm.

(18) a(t)=Φq(t),

where Φ is the stator modal shape normalized to the mass matrix M. Then, Equation (20) can be transformed into modal space as

(19) $$\ddot{q}\left(t\right)+\left(\alpha I+\beta \Lambda^{2}\right)\dot{q}\left(t\right)+\Lambda^{2}q\left(t\right)=\Phi^{T}\left[F^{*}\left(t\right)+F_{pre}+F_{N}\left(\tilde{a},a\left(t\right)\right)+F_{T}\left(\tilde{a},a\left(t\right),\dot{a}\left(t\right),{\dot{u}}_{m}\left(t\right)\right)\right],$$

where α and β are Rayleigh damping constants calculated from modal damping ratios $\xi_{i}$, and $\xi_{i}$ is the ratio damping for the i th mode shape. $\Lambda^{2}$ is a diagonal matrix containing the square of natural circular frequency $\omega_{i}^{2}$ on the diagonal [30]. Because the stator of an LUM usually drives the mover at particular modes of vibration and the non-operating natural frequencies are designed to be far away from the operating frequencies, only two modes of vibration, the symmetric mode $\varphi_{1}$ and the asymmetric mode $\varphi_{2}$, are considered (i.e., $\Phi =[\begin{array}{cc} \varphi_{1} & \varphi_{2} \end{array}]$) for the calculation in model space. Then the high-dimensional nonlinear system is reduced to a two-dimensional system which can be solved efficiently. The dynamic frictional contact process and the output performance of the mover can be simulated by solving the mover model in Equation (3) and the stator model in Equation (19) simultaneously. To ensure the calculation precision, the explicit ODE45 solver based on the Runge–Kutta integration scheme is used to simulate this problem in MATLAB [31]. Figure 6 shows the main steps of the algorithm for analyzing the dynamic stator–mover frictional contact.

It should be noted that the state of the stator after applying pre-pressure is set to the initial condition and the pre-pressure is assumed to be a constant value. The input variables are the electric excitation signal applied to PZT, pre-pressure force, statistical micro-topography parameters of contact interfaces, and external load applied to the mover. The dynamic response of the stator and mover, including displacements, velocities, and the frictional contact forces of all nodes at the contact tip surface, can be obtained based on the model.

## 4. Measurement System of Stator–Mover Frictional Contact Forces

In this section, the method to simultaneously and quantitatively measure the dynamic normal and tangential contact forces between the stator and mover in LUM is presented, which is important for verifying the simulation results and modifying some input parameters in contact algorithms appropriately. A piezoelectric bimorph sensor was designed based on the longitudinal and shear direct piezoelectric effects [32], and it was patched to the surface of the friction bar for measuring the dynamic normal and tangential contact forces at high frequency. The feasibility of this measurement method is validated by finite element simulation [33]. The relationship between the output voltages of the piezoelectric sensor and the frictional contact forces is calibrated experiment. Then the dynamic frictional contact forces can be obtained by measuring the voltages of the sensor. The measurement principle and system will be described in detail.

### 4.1. Measurement Principle

**Figure 7:**
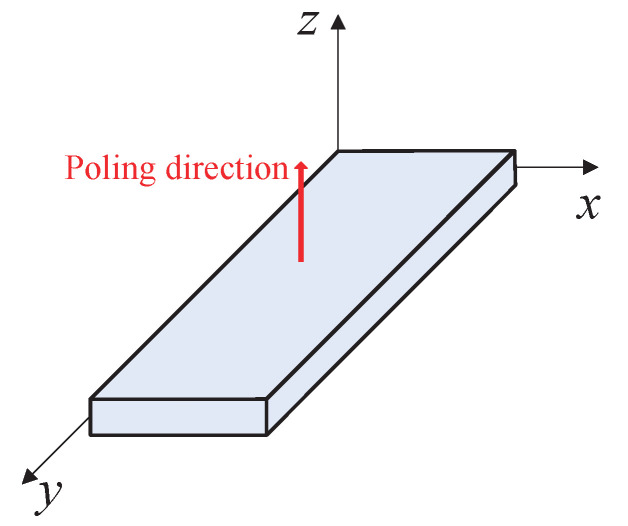
Schematic of a piezoelectric element.

According to the theory of piezoelectricity [34,35,36], when the external electric field is zero, the constitutive relations for a piezoelectric element shown in Figure 7 can be expressed as:

(20) $$\left[\begin{array}{c} D_{1}\\ D_{2}\\ D_{3} \end{array}\right]=\left[\begin{array}{cccccc} 0 & 0 & 0 & 0 & d_{15} & 0\\ 0 & 0 & 0 & d_{24} & 0 & 0\\ d_{31} & d_{31} & d_{33} & 0 & 0 & 0 \end{array}\right]\left[\begin{array}{c} T_{11}\\ T_{22}\\ T_{33}\\ T_{23}\\ T_{31}\\ T_{12} \end{array}\right],$$

where $D_{1},D_{2},D_{3}$ represent the electric displacement in directions x,y,z. $T_{11},T_{22},T_{33}$ represent the normal stress applied along directions x,y,z, which is called transverse or longitudinal direct piezoelectric effect. $T_{23},T_{31},T_{12}$ represent the shear stress in planes yz,zx,xy, which is called shear direct piezoelectric effect. Note that all of the normal stresses only contribute to the electric displacement in direction *z*, and the shear stress in yz and zx planes only contribute to the electric displacement in *y* and *x* directions, respectively. For an operating LUM, the stator–mover contact behavior will generate normal contact force and tangential shear force. Moreover, the electric displacement is strictly proportional to the generated potential differences ($V_{1},V_{2},V_{3}$) in the corresponding two opposing faces of the piezoelectric element, and independent of the piezoelectric element size and shape [34]. The potential difference between two electrodes on the piezoelectric element surface is easily measured. Just know the relationship between the measurement potential difference and the applied force, which would be given by simulation and experiment in a later section, then the corresponding force under different operational modes of piezoelectric can be measured.

**Figure 8:**
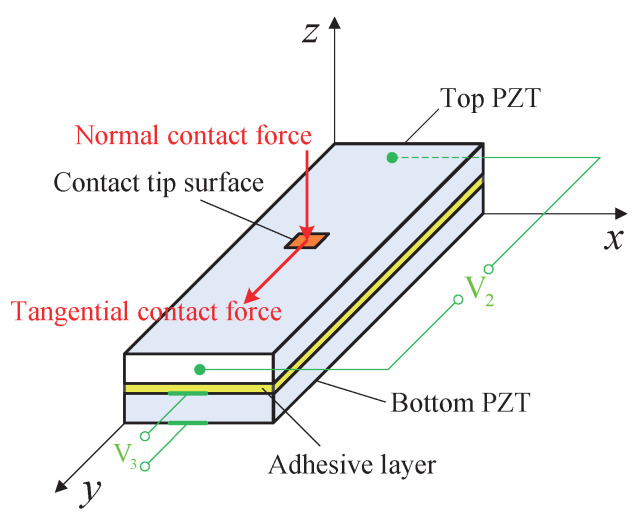
Schematic of the piezoelectric sensor used to measure frictional contact forces.

Based on the above analysis and principle, the frictional contact forces applied by the stator to the mover can be measured by patching two piezoelectric elements to the friction bar. The longitudinal direct piezoelectric effect is designed to get the normal contact force by measuring the potential differences in the *y* direction, and the shear direct piezoelectric effect is designed to get the tangential contact force. Figure 8 shows the schematic of the designed piezoelectric sensor for measuring the frictional contact forces simultaneously. Two PZT-5H piezoelectric ceramics with the same thickness of 0.5 mm and the same poling directions are bonded up and down by epoxy. The piezoelectric element used to measure the potential difference $V_{3}$ generated by the normal contact force is located bottom to avoid contacting the contact tip of the stator directly because its two faces in *z* direction need to be coated with thin electrode layer. The piezoelectric element located on top is used to measure the potential difference $V_{2}$ generated by the tangential contact force, and its two opposing faces in *y* direction are coated with an electrode layer so it can directly contact the stator. This arrangement of piezoelectric elements is to guarantee the measurement accuracy of tangential contact force and the transmission loss of normal contact force is neglected. Therefore, the thickness of epoxy between two piezoelectrics should be thin enough to satisfy the insulation between two piezoelectric elements.

### 4.2. Feasibility Analysis

**Figure 9:**
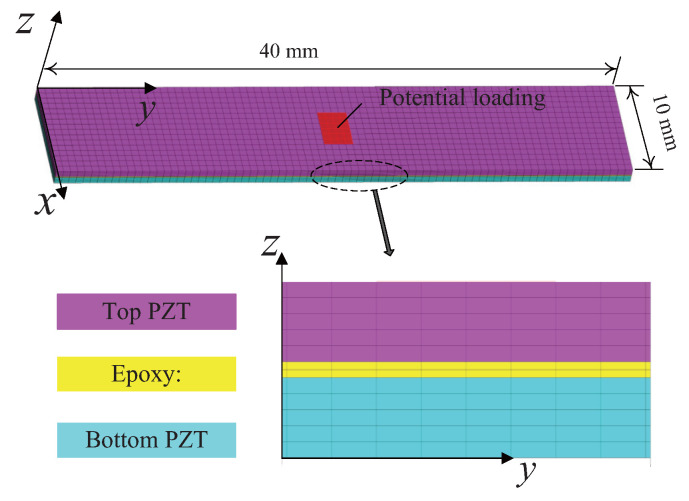
Finite element mesh of the piezoelectric bimorph sensor.

For validating the feasibility of the measurement method, finite element simulations are performed first to simulate the electric potential distribution of the piezoelectric bimorph sensor under static normal and tangential forces. Figure 9 shows the 3D finite element mesh of this piezoelectric sensor. The thickness of the epoxy adhesive layer is set at 0.1 mm. The material parameters of PZT-5H and DP460 epoxy in [37] are employed. A total of 17,901 nodes and 15,384 isoparametric cubic elements with 8 nodes are used for the whole model, and the maximum mesh size of 0.5 mm is taken. The fixed displacement constraint is applied to the bottom of the piezoelectric sensor. The grounding electrode is set for the left surface of the top piezoelectric element and for the bottom surface of the bottom piezoelectric element. Then the electric potential on the right surface of the top piezoelectric element $V_{2}$ and on the top surface of the bottom piezoelectric element $V_{3}$ will be the output voltages. The frictional contact forces are applied in the form of surface force, and the size of the contact tip surface is set as 3×2${\mathrm{mm}}^{2}$ which is the same as the actual area of the contact tip surface. In this case, the finite element equations in matrix form can be written as

(21) $$K_{s}\left[\begin{array}{c} u_{s}\\ V_{2}\\ V_{3} \end{array}\right]=F_{p},$$

where $K_{s}$ is the stiffness matrix of finite element sensor model, and $u_{s}$ is the vector of unknown DOF values except the measuring electric potential $V_{2}$ and $V_{3}$. $F_{p}$ is the equivalent nodal force vector of potential loading including normal and tangential contact forces.

**Figure 10:**
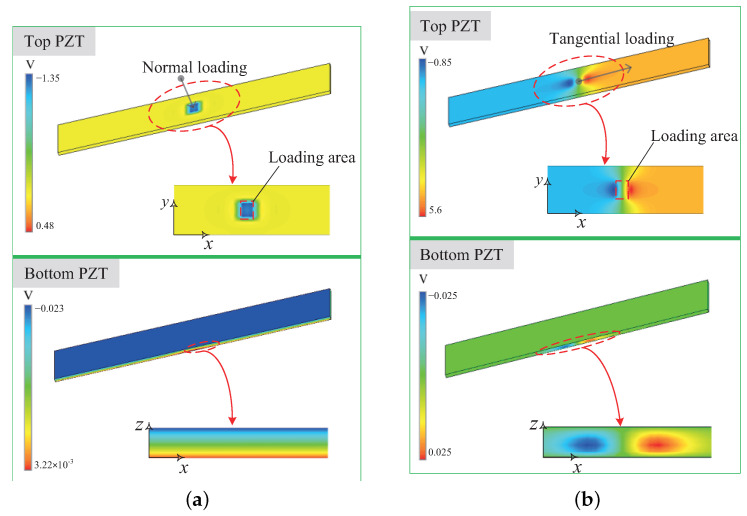
Electric potential distribution of sensor under: (**a**) unit normal force; (**b**) unit tangential force.

When a normal loading of 1 N is applied to the sensor, the distribution of electric potential in the bottom and top piezoelectric element is plotted in Figure 10a. The simulation results are basically inconsistent with the theoretical analysis. The bottom PZT generates an electric potential difference between the two opposing faces in direction *z*. The top PZT also exists electric potential variation but only near the loading area, which does not affect the electric potential difference between the two opposing faces in direction *x*. When a tangential loading of 1 N is applied to the sensor, the distribution of electric potential for two piezoelectric elements is plotted in Figure 10b. The top PZT generates an obvious electric potential difference between the two opposing faces in direction *x*, and there is an electric potential concentration near the loading area. Although the bottom PZT generates electric potential change near the loading area, the electric potential between its up and down surfaces remains the same, i.e., the tangential loading does not affect the output voltage of the bottom PZT. It can be seen that as long as the loading position avoids the edge of the sensor, the output voltage of the bottom PZT only relates to the normal loading acting on the sensor surface and the output voltage of the top PZT only relates to the tangential loading.

**Figure 11:**
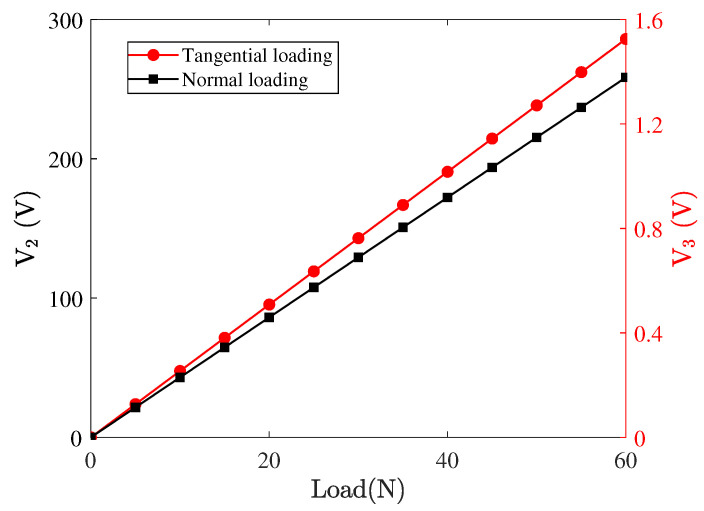
The force–voltage relationship for the designed sensor.

Furthermore, the sensor output voltages ($V_{3}$ and $V_{2}$) are simulated when different static normal and tangential forces are, respectively, applied to the central position of the sensor. Figure 11 shows the variation of output voltage $V_{2}$ as the value of tangential loading $F_{T}$ increases from 0 N to 60 N, and the variation of output voltage $V_{3}$ with the normal loading $F_{N}$. It can be seen that the tangential and normal loading applied to the sensor are proportional to the output voltages of the top and bottom PZT, respectively. Based on the simulation results, the tangential and normal loading acting on the sensor can be expressed linearly as a function of the two output voltages of the sensor, which are $F_{T}=0.21V_{2}$ and $F_{N}=43.5V_{3}$. Considering the roughness of contact interfaces, the actual contact area is less than the nominal area of the contact tip which may affect the output voltages of the sensor. Different sizes of loading areas were also used to simulate the relationship. The identical numerical relationships between loadings and voltages were obtained, which will not repeat here, i.e., the measured voltage only depends on the values of frictional contact forces. To sum up, the accurate measurement of the tangential and normal contact forces acting on the sensor is feasible by monitoring the two output voltages of the sensor, as long as the contact tip is away from the two ends of the sensor.

### 4.3. Experimental Calibration

**Figure 12:**
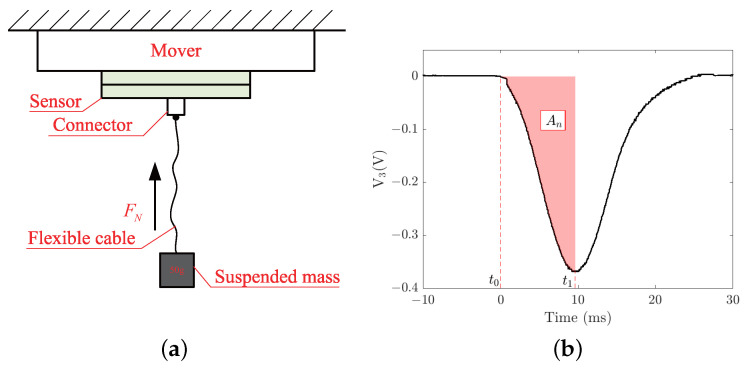
Normal calibration of the sensor: (**a**) schematic diagram of experimental scheme; (**b**) first output voltage waveform of the bottom PZT.

Based on the static simulation analysis of the sensor model, both the tangential and normal forces applied to the sensor are considered to be proportional to its output voltages $V_{2}$ and $V_{3}$, and the output voltages only dependent on the loading value and not the loading area. Combined with practical analysis, the sensor prototype was produced and a calibration experiment platform was built to calibrate the relationships between the potential loading applied to the sensor and the output voltages. Figure 12a shows the experimental schematic diagram for calibrating the relationship between the normal loading applied to the sensor surface and output voltage $V_{3}$. After bonding the sensor to the contact surface of the mover, a mass of 50 g is hung on the sensor by a flexible cable and a connector bonded to the sensor surface. By dropping the suspended mass vertically to generate normal disturbance to the sensor, the bottom PZT of the sensor would output a voltage signal and the first waveform is recorded by an oscilloscope. When the falling height *h* of the mass is 10 cm, the first output voltage waveform of the bottom PZT is approximately a half sinewave, as shown in Figure 12b. According to the momentum principle, the momentum change of mass from $t_{0}$ to $t_{1}$ is equal to the impulse of the pulling force from the cable during this time, which can be expressed mathematically as

(22) $$p_{1}-p_{2}=\int_{t_{0}}^{t_{1}}F_{n}dt,$$

where $p_{1}=m_{s}\sqrt{2gh}$ is the momentum of suspended mass at $t_{0}$. The momentum $p_{2}$ at $t_{1}$ is zero because the pulling force of the cable reaches a maximum value and the velocity of the mass is zeros. $g=10\;m/s^{2}$ is the gravitational acceleration, and $m_{s}$ is the mass of the suspended mass. If the relationship between normal loading $F_{n}$ and output voltage $V_{3}$ is assumed as $F_{n}=c_{n}V_{3}$, then the coefficient $c_{n}$ can be obtained by

(23) $$c_{n}=\frac{m_{s}\sqrt{2gh}}{A_{n}},$$

where $A_{n}=\int_{t_{1}}^{t_{2}}V_{3}dt$ is the shaded areas between voltage signal and time axis from $t_{1}$ to $t_{2}$.

**Figure 13:**
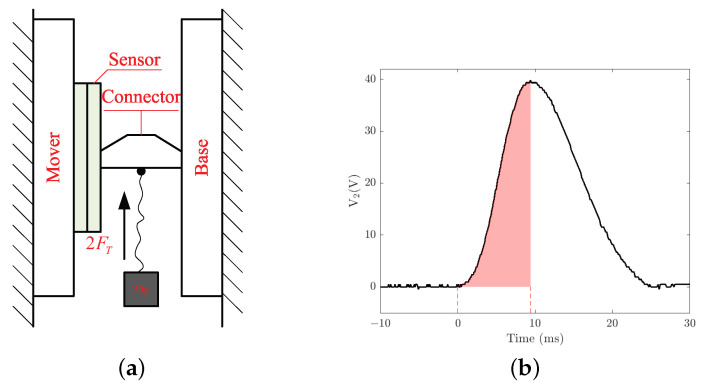
Tangential calibration of the sensor: (**a**) schematic diagram of experimental scheme; (**b**) first output voltage waveform of the top PZT.

Similarly, Figure 13a shows the experimental schematic diagram for calibrating the relationship between the tangential loading applied to the sensor surface and output voltage $V_{2}$. When the suspended mass is dropped vertically, a tangential disturbance would be applied to the sensor. Figure 13b shows the first output voltage waveform of the bottom PZT when the falling height *h* of the mass is 10 cm. It is noted that only half of the force of the cable is transferred to the sensor surface, i.e., the output voltage $V_{2}$ is only half of the force on the cable. If the relationship between tangential loading $F_{t}$ and output voltage $V_{2}$ is assumed as $F_{t}=c_{t}V_{2}$, the coefficient $c_{t}$ can be calculated by

(24) $$c_{t}=\frac{m_{s}\sqrt{2gh}}{2A_{t}}.$$

In order to ensure the accuracy of the experiment, five sets of falling heights h=10, 15, 20, 25, and 30 cm were chosen for identifying the two coefficients $c_{n}$ and $c_{t}$, as listed in Table 1. It can be seen that the two coefficients obtained by the experiment coincided with the simulation result better for higher falling heights, which may be due to the reduced proportion of the output voltage signal caused by environmental interference. So the two conversion factors ($c_{n}=43.5$ and $c_{t}=0.21$) are used to convert the output voltage of the sensor into the normal and tangential forces applied to the sensor.

### 4.4. Measurement System

**Figure 14:**
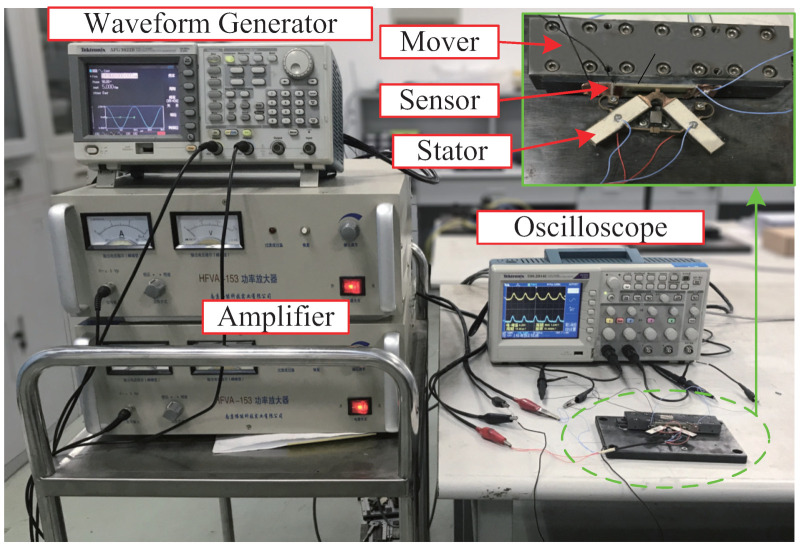
Measurement system for the stator–mover dynamic contact.

Figure 14 shows the measurement system for measuring the dynamic frictional contact between the stator and mover. A wave generator (AFG 3022B, Tektronix, Inc., Beaverton, OR, USA) is used to generate two electronic signals with special amplitude, frequency, and phase difference, which is output to an amplifier (HFVA-42, Nanjing Funeng Technology Corp., Nanjing, China). Then the two amplified electronic signals are applied to the stator of the LUM. Dynamic friction contact will happen between the sensor bonded to the mover and the contact tip. With the action of normal and tangential contact forces, the top and bottom PZT of the sensor would output corresponding voltages which are measured by a digital oscilloscope (TDS 2014C, Tektronix, Inc., USA) with storage capability. The normal and tangential contact forces can be calculated through the simulated calibration of the sensor by the finite element method.

## 5. Experimental Validation and Numerical Simulation

Based on the developed numerical model and measurement system for the stator–mover contact issue in LUM, some static and dynamic contact behaviors between the stator and mover, and some output performances of the motor are simulated, measured, and discussed. The material parameters of $Al_{2}O_{3}$ ceramic and manganese steel are employed for the friction bar and substrate of the stator, respectively. The parameters used in numerical simulations are listed in Table 2 unless stated otherwise.

### 5.1. Static Stator–Mover Contact

It is well-known that pre-pressure is an important factor affecting the output performance of the motor. Because the state-mover contact state is an initial condition for motor operation and the initial contact force between the stator and mover would affect the driving force of the contact tip to the mover. In addition, the stator–mover contact state under pre-pressure is also the foundation for conducting the dynamic contact algorithm because the pre-pressure in the dynamic model is characterized by the static penetration depth ${\tilde{u}}_{i}$. Therefore, investigation of the static stator–mover contact behavior of LUM under pre-pressure is necessary.

**Figure 15:**
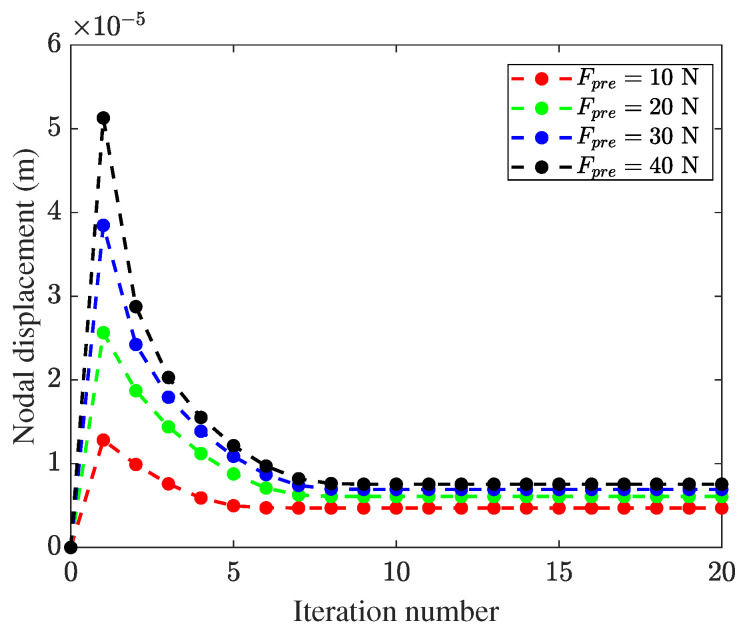
Nodal displacement monitor in an iterative process.

Firstly, the convergence of the static contact algorithm is investigated for different pre-pressures. Figure 15 shows the displacement monitor chart of the center node at the contact tip surface as the iterative number increases, where the pre-pressures $F_{pre}=10,20,30,40$ N are chosen. It can be seen that the monitored nodal displacement converges at an exponential rate and the results meet the required accuracy in ten iterations.

**Figure 16:**
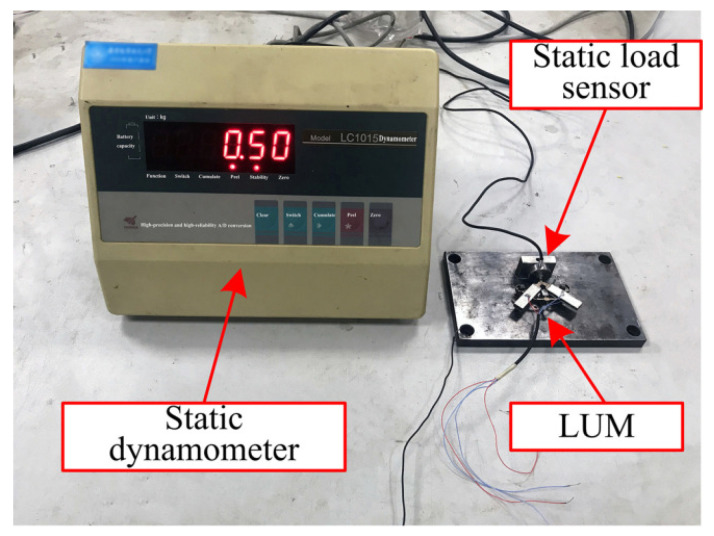
Experimental setup for measuring static contact force.

**Figure 17:**
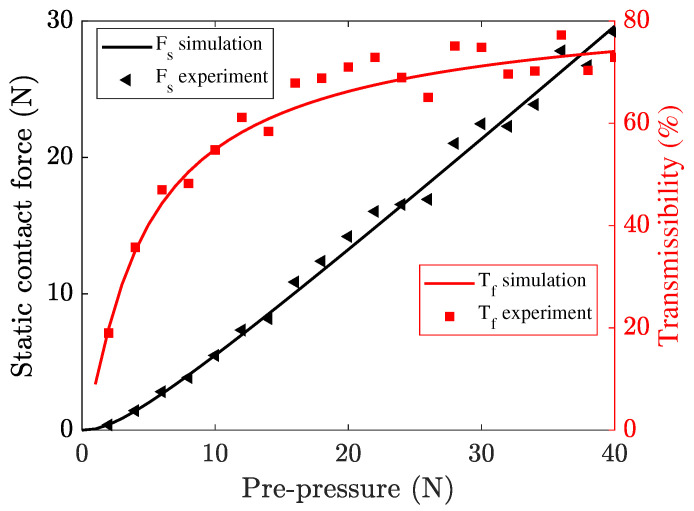
Experimental and simulation results for static contact force.

**Figure 18:**
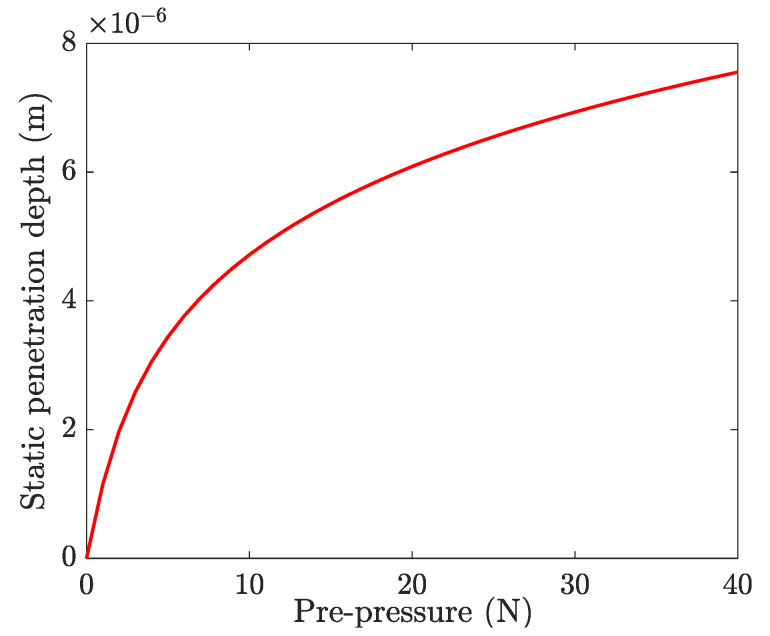
Variation of a nodal displacement with pre-pressure.

Subsequently, the actual static contact forces for different pre-pressure were measured by a static dynamometer (LC1015, Lance Technology Inc., Copley, OH, USA), as shown in Figure 16. The static contact forces ($F_{s}$) as the pre-pressure from 0 to 40 N were measured and calculated. Figure 17 shows the variation of the static contact force with the pre-pressure, as well as the transmissibility of pre-pressure ($T_{f}$) defined by the ratio of contact force to pre-pressure. As the pre-pressure increases, both experimental and simulation results show that the transmissibility of pre-pressure increases rapidly and reaches a stable value of about 74%. The roughness at contact interfacial is thought as the most possible reason for this phenomenon because only a small part of asperities come into contact when the pre-pressure is relatively small and then the structure of the stator would bear more load from the pre-load spring. This means that the roughness of contact surfaces cannot be ignored for accurate simulation, especially when the pre-pressure is small. The static displacements for all of those nodes at the contact tip surface also were output and used as the static penetration depth ${\tilde{u}}_{i}$ in the dynamic contact algorithm. Figure 18 shows the variation of a nodal displacement with the pre-pressure, which illustrates a nonlinear relationship when considering the interfacial roughness.

### 5.2. Dynamic Stator–Mover Contact

#### 5.2.1. Validation of the Dynamic Contact Algorithm

The proposed quantitative measurement method in Section 4 for the dynamic frictional contact forces between stator and mover is used to check the quality of the frictional contact model and the dynamic contact algorithm presented in Section 3. In order to minimize the experiment errors, the mover was fixed to guarantee that the stator–mover contact condition remains unchanged as possible, and the interfacial wear was ignored. In this case, the corresponding simulation was conducted by setting the mover velocity as zero in the dynamic contact algorithm. The time evolutions of normal and tangential contact forces were compared between experimental and numerical results in the stable phase when the LUM was operating under different pre-pressures and exciting voltages.

**Figure 19:**
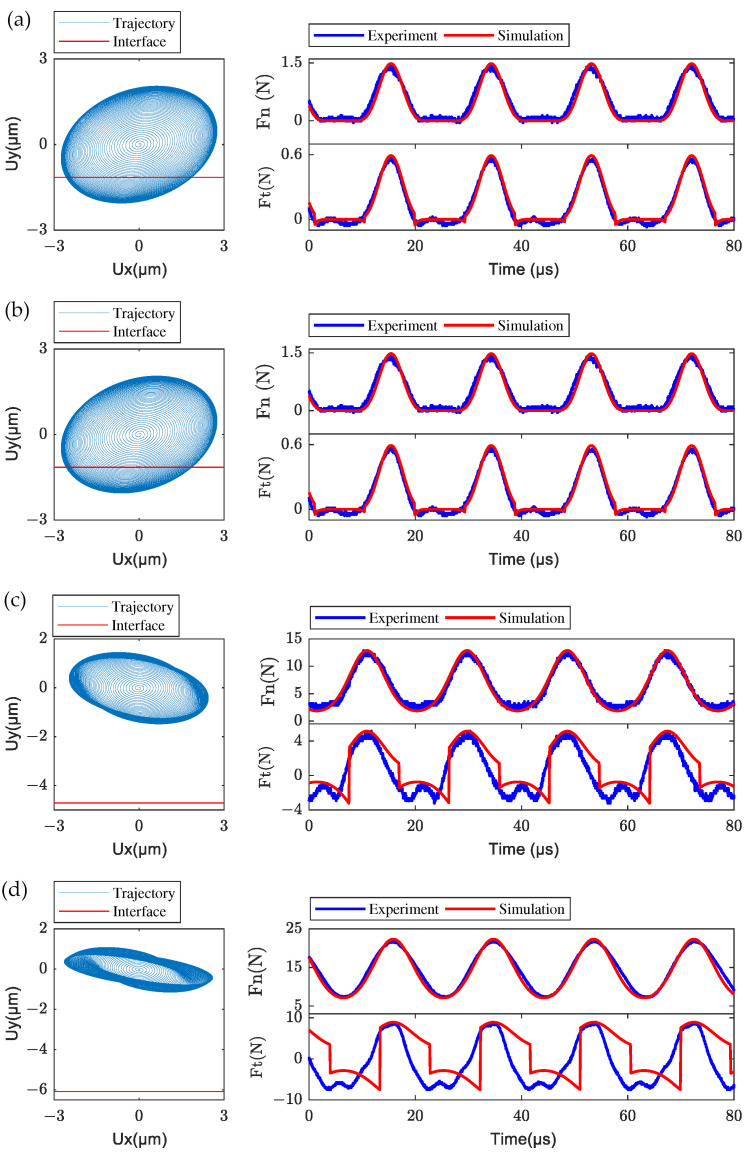
Transient nodal trajectory at the center of the contact tip and the comparison of frictional contact forces between experiments and simulations for different pre-pressures: (**a**) 0 N, (**b**) 1 N, (**c**) 10 N, and (**d**) 20 N.

With the exciting voltage of 50 V and frequency of 53 kHz, transient numerical simulations of the LUM with fixed mover were conducted from 0 to 3 ms when $F_{pre}=0,1,10,20$ N were chosen. By numerical integration of Equation (19), the transient vibration of the stator in modal space was obtained. Then the transient nodal displacements of the contact tip were calculated by Equation (18), and the normal contact force of each node is given by the developed normal contact model. The nodal trajectory of the central node of the contact tip surface in plane xy is extracted and shown in Figure 19, as well as the comparison of measured and simulated frictional contact forces, including normal contact force (Fn) and tangential frictional force (Ft), during 80 μs of the steady-state phase, was carried out. Ux and Uy denote the displacement of this node in the x direction and y direction, respectively. The normal and tangential contact forces evolve periodically with time in both experimental and simulated results, and the response frequency of the dynamic frictional contact forces is the same as the frequency of the electric excitation. The measured normal and tangential contact frictional forces match the results of numerical simulation well for low pre-pressures (e.g., $F_{pre}=0,1$ N). The intermittent contact behavior between the stator and mover can be characterized obviously in both experimental and simulated results, where the frictional contact forces vanished when the stator separated from the mover. For the relatively high pre-pressures (e.g., $F_{pre}=10,20$ N), the assumed penetration depth in simulations would also be larger as is shown by the nodal transient trajectory for different pre-pressure, which may lead to greater simulation error of the tangential contact force. It also can be seen that a discontinuous tangential contact force occurs in simulation results with a relatively high pre-pressure, which generally exists in the contact model describing the stator–mover contact of LUM [7,8,11]. This is because of the discrete time in simulations which results in a discontinuous tangential velocity that relates to the tangential contact force by the regularization friction model. Despite some values of frictional contact forces being missing, it is clear that the changing trend and the amplitude of simulated contact forces also agree with the experimental results, i.e., the dynamic frictional contact model is still effective for investigating the dynamic frictional contact mechanism between stator and mover when the pre-pressure is high.

**Figure 20:**
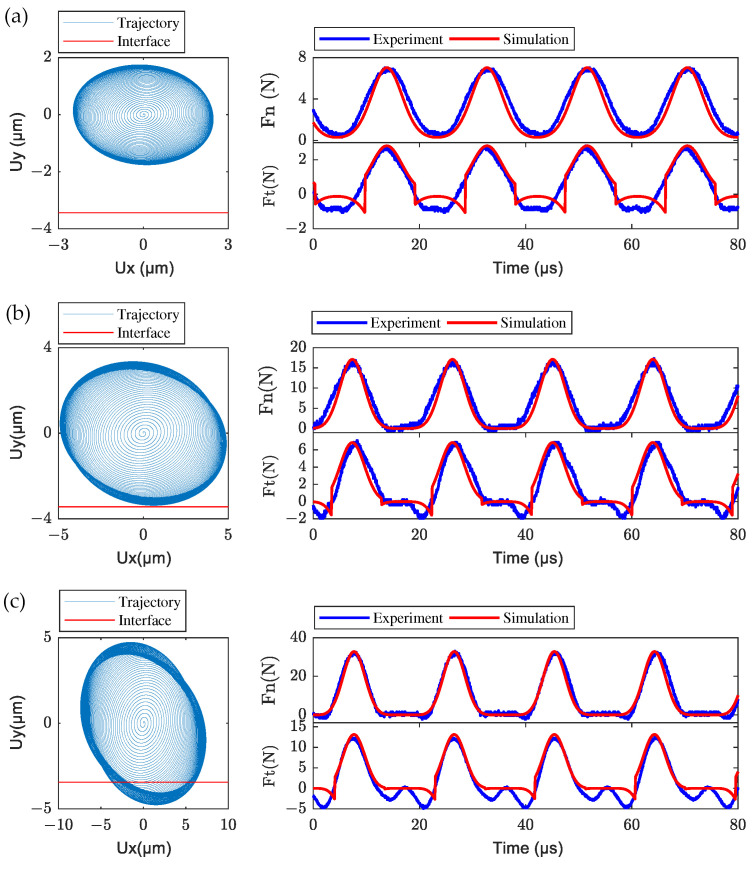
Transient nodal trajectory at the center of the contact tip, and the comparison of frictional contact forces between experiments and simulations for different exciting voltages: (**a**) 50 V, (**b**) 100 V, and (**c**) 150 V.

With the fixed pre-pressure of 5 N, transient simulations of the LUM with the fixed mover were conducted when the exciting voltages of 50, 100, and 150 V were chosen. The simulated transient trajectory of the node located at the center of the contact tip surface is given, and the corresponding stable frictional contact forces between stator and mover were measured by experiment and compared with simulated results, as shown in Figure 20. From the nodal transient trajectory, the amplitude of the stator vibration in *x* and *y* directions increases with the increasing of exciting voltage, and there is a separate phase between the stator and mover when the exciting voltage is 150 V. From the experimental and simulated contact forces in the stable state, the separation between stator and mover could also be characterized by the vanishing of the normal contact force. In the experimental result, the negative tangential contact force also remains when the normal contact force is zero (i.e., during separate phase), which may be caused by the transverse shear effect of the two rough contact interfaces. Specific reasons await our further study. From all these comparisons between simulation and experimental results, the presented dynamic contact algorithm is effective for analyzing the stator–mover contact mechanism and the presented measurement system can be used to measure the dynamic contact forces between the stator and mover in LUM quantitatively.

#### 5.2.2. Transient Analysis of the Force Transmission at Contact Interfaces

**Figure 21:**
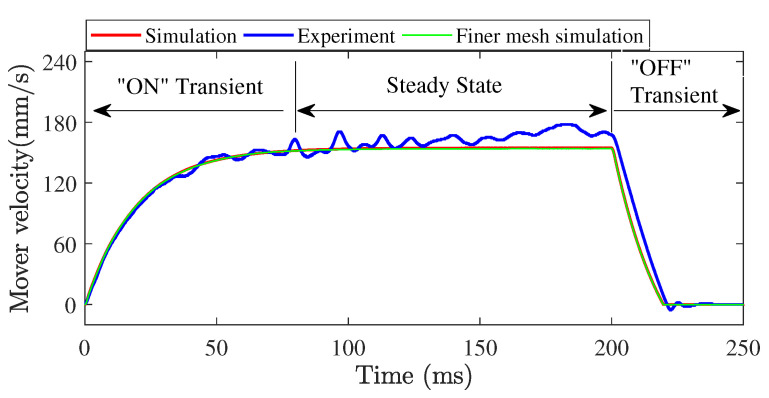
Comparison between simulation and experiment results of the mover transient velocity.

In order to clearly understand the frictional driving mechanism of LUM, a complete operational process, from startup to steady state and powered off at 200 ms, is simulated. The exciting voltage and pre-pressure are 100 V and 5 N, respectively. Considering that the mesh quality of the contact surface may affect the simulation results of the frictional contact forces, a stator model with a mesh size of 0.25 mm for the contact surface is also used. By simultaneously solving the stator model in Equation (19) and the mover model in Equation (3), the stator vibration and mover motion were output. Then the normal contact force was calculated by the nodal displacements on the contact tip surface in the normal contact model, and the tangential friction force was calculated by the nodal relative velocity between the nodes on the contact tip surface and mover. Figure 21 shows the time evolution of the mover velocity obtained by experiment and simulation. The good agreement between experiment and simulation further verifies the effectiveness of the developed frictional contact model. The velocity fluctuation in experiment results may be because the mover is not an absolutely ideal single-degree-freedom system or the mover contact interface is position-dependent due to insufficient manufacture accuracy. With the motion of the mover, the variation of driving conditions would lead to the change of frictional contact forces, then the velocity of the mover would be changed. That is also why the mover is fixed in the experimental validation of the dynamic contact algorithm. Additionally, the model with the finer mesh for the contact surface takes three times as long to complete the simulation; however, the maximum velocity error simulated by the two models with different mesh sizes for the contact surface is lower than 0.7%, which means that the mesh size of 0.5 mm for contact surface has been able to meet the simulation accuracy and take up less computational cost. According to the dynamic variation process of the mover velocity, the whole process is divided into the ON transient stage, steady-state stage, and OFF transient stage. It can be seen that the velocity of the mover achieves stability at about 80 ms after start-up, and fast decays to zeros in about 20 ms after power-off due to friction self-locking.

**Figure 22:**
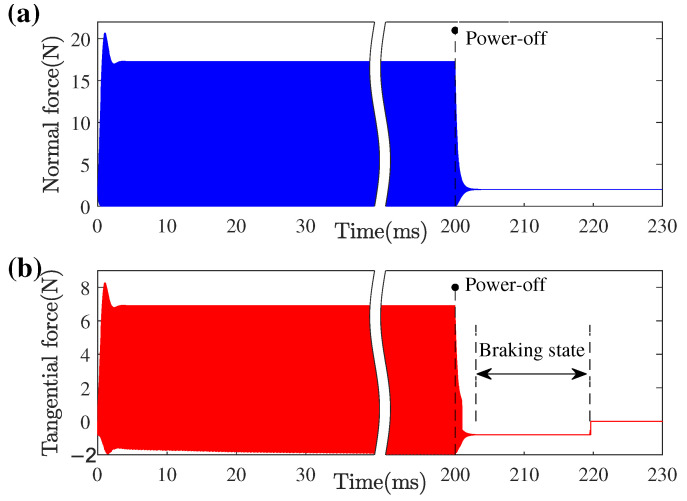
Transient forces transmission of contact interfaces: (**a**) normal contact force; (**b**) tangential contact force.

In this case, the time-dependent normal and tangential forces transferred to the mover are calculated, as shown in Figure 22. Different from the variation of mover velocity, the frictional contact force transferred between the stator and mover is stable for only several milliseconds after start-up, which indicates that the stator vibration reaches a steady state. The slight change of tangential contact force is due to the increase of mover velocity and the periodic driving mechanism. Under the stable driving of the contact tip, the mover velocity continues to increase until a dynamic balance of the whole system is obtained. After power-off, the normal force drops to the static normal contact force under the application of pre-pressure in 4 ms. The tangential force first stabilizes at a negative value, which is called the braking state, and then vanishes at the time when the mover velocity is zero. This indicates that the stator first stops vibration and acts as a brake through the friction forces until the velocity of the mover drops to zero, which is also the self-locking principle of the LUM.

**Figure 23:**
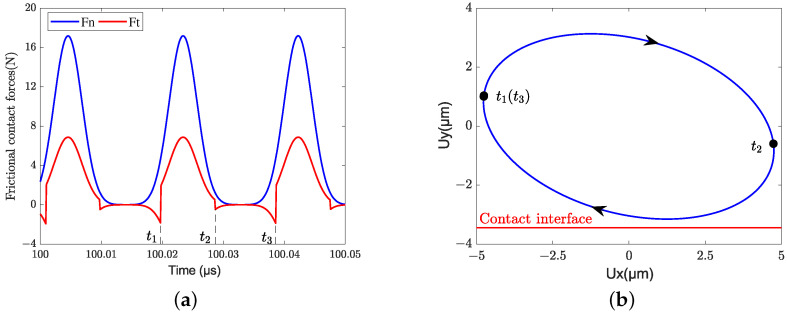
Steady-state driving mechanism of LUM: (**a**) contact forces; (**b**) driving trajectory.

Although the stator–mover frictional contact behavior is strongly nonlinear, the interfacial contact forces and the driving trajectory of the contact tip still change periodically in the simulation results. Figure 23 shows the interfacial contact forces of one full driving cycle (from $t_{1}$ to $t_{3}$) during steady-state velocity and the corresponding elliptical trajectory of the central node at the contact tip is given to analyze the driving mechanism. It can be seen that the tangential contact force (i.e., the driving force of the mover) is positive from $t_{1}$ to $t_{2}$; however, it is negative from $t_{2}$ to $t_{3}$. This is because the motion of the contact tip is a back-and-forth movement, however, the mover is always moving in one direction. When the relative velocity between the contact tip and the mover is greater than zero, the contact tip would do positive work for the mover. Otherwise, the contact tip would do negative work for the mover. It should be noted that there is a time period from $t_{2}$ to $t_{3}$ where both the normal and tangential forces are almost zero. In this time period, only a few asperities are involved in contact and the mover keeps moving forward mainly by inertia. If the pre-pressure is decreased or the excitation voltage is increased, the contact tip would separate the mover during this time period.

**Figure 24:**
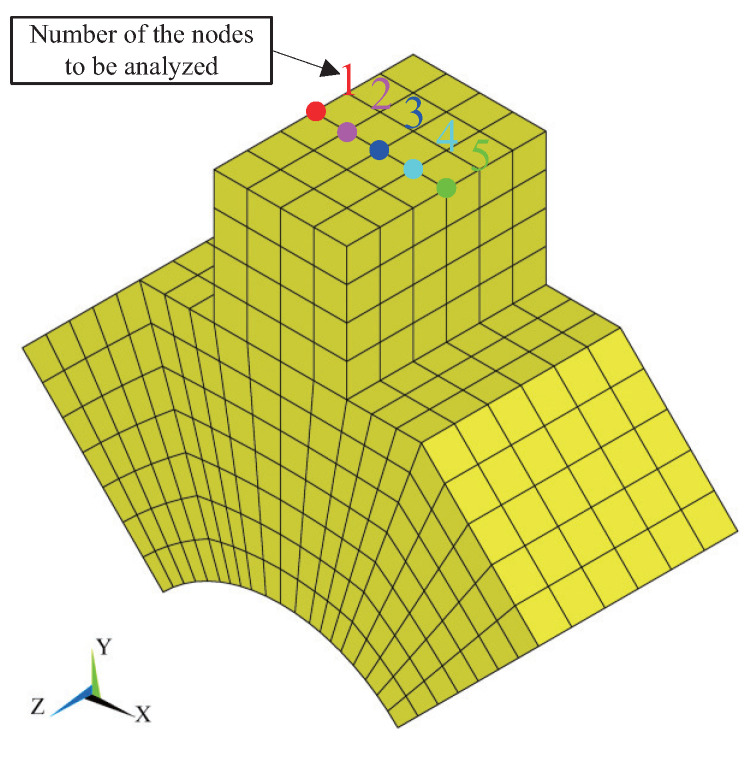
Distribution of the five analyzed nodes at the contact interface.

**Figure 25:**
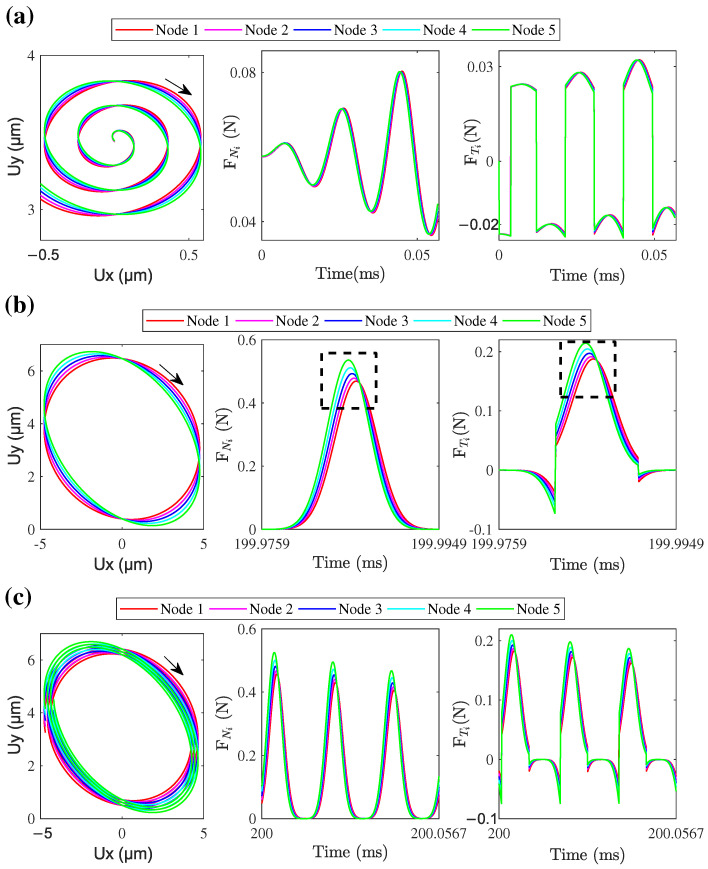
Simulated trajectories and contact forces for 5 nodes at the contact surface: (**a**) first three circles in the ON transient state, (**b**) one circle in steady state, and (**c**) first three circles in the OFF transient state.

**Figure 26:**
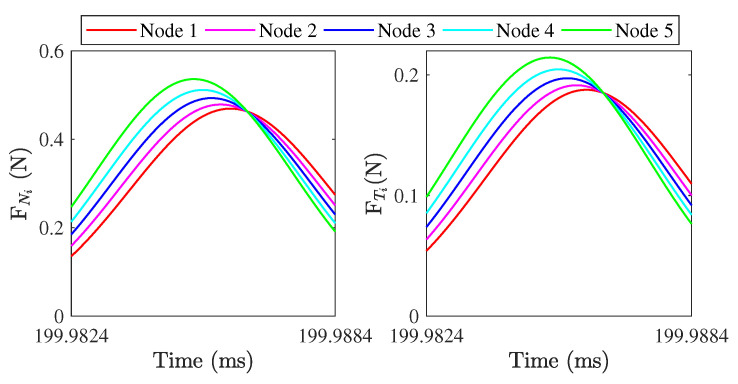
Partially enlarged view of the dashed area in Figure 23a.

To investigate the force transmission of different positions at the contact tip surface, five nodes along *x* direction are selected for the analysis of nodal load transmission, as is shown in Figure 24. Because the operation of this motor is based on the vibration in xy plane, only the nodal forces transferred in *x* and *y* directions are analyzed and the contact state of nodes in *z* direction is considered identical. Figure 25 shows the nodal trajectories and carried forces of the first three cycles during the ON transient state, one cycle during the steady state, and the first three cycles during the OFF transient state. It can be seen that the simulated trajectories of the five nodes are different during all three states. During the ON transient state, the difference of contact forces is not obvious for these nodes and both the normal and tangential contact forces demonstrate the characteristic periodically increasing behavior. It is interesting that the proportion of positive and negative tangential force in time is almost equal during one cycle. However, when the value of the positive amplitude is greater than the value of the negative amplitude, the difference in work between positive and negative tangential forces drives the mover to accelerate. During the steady state and OFF transient state, the carried forces at different nodes show hysteresis in time, as well as their amplitudes, are different. According to the partially enlarged view of the dashed area in Figure 25b, as shown in Figure 26, the carried peak forces of these nodes along *x* direction decrease from node 5 to node 1, and the tangential and normal forces of node 5 first reach the peak value due the clockwise motion of contact tip. Node 5 has 12 percent more force transmission than node 1. Obviously, the wear of different positions at the contact tip surface would be different. Obtaining the multi-point force transmission at the contact interfaces would be useful for analyzing the interfacial wear of the LUM. When the electric excitation is powered off, the interfacial transferred forces will decay periodically and the attenuation rate is about 5.5%.

#### 5.2.3. Energy Transmission at Contact Interfaces

According to the above analysis, the possible energy transmission between the stator and mover includes the positive driving energy, the negative driving energy, as well as the frictional loss energy due to the slip of the stator–mover contact interfaces. Due to the periodic force transmission process between the contact tip and the mover, the three energies acted by the contact tip to the mover are calculated in terms of period. Because the numerical simulation results are discrete in time, one running cycle is divided into a series of time steps. The energy transmission of one node during a single time step can be approximated as

(25) $$\left\{\begin{array}{c} W_{ij}=F_{{}_{T_{ij}}}{\dot{u}}_{m_{j}}\delta t\\ W_{ij}^{*}=F_{{}_{T_{ij}}}v_{ij}\delta t \end{array}\right.$$

where *i* and *j* are the numbers of interfacial nodes and time steps. *v* is the relative velocity between the node and mover. $W_{ij}$ is the work done by one contact node to the mover, and $W_{ij}^{*}$ is the energy loss due to sliding friction. Then the positive driving, negative driving and frictional loss energies ($W_{p}$, $W_{n}$, and $W_{f}$) of all nodes during one running cycle can be given by

(26) $$\left\{\begin{array}{c} W_{p}=\sum_{i}\sum_{j}\left(W_{ij}^{+}\right)\\ W_{n}=\sum_{i}\sum_{j}\left(W_{ij}^{-}\right)\\ W_{f}=\sum_{i}\sum_{j}\left(W_{ij}^{*}\right) \end{array}\right.$$

Here, $W_{ij}^{+}$ and $W_{ij}^{-}$ donate the elements greater and less than zero in $W_{ij}$, respectively. The proportions of the three energies in one cycle are given by

(27) $$\left\{\begin{array}{c} \lambda_{p}=\frac{W_{p}}{W_{p}+\left|W_{n}\right|+W_{f}}\\ \lambda_{n}=\frac{\left|W_{n}\right|}{W_{p}+\left|W_{n}\right|+W_{f}}\\ \lambda_{f}=\frac{W_{f}}{W_{p}+\left|W_{n}\right|+W_{f}} \end{array}\right.$$

**Figure 27:**
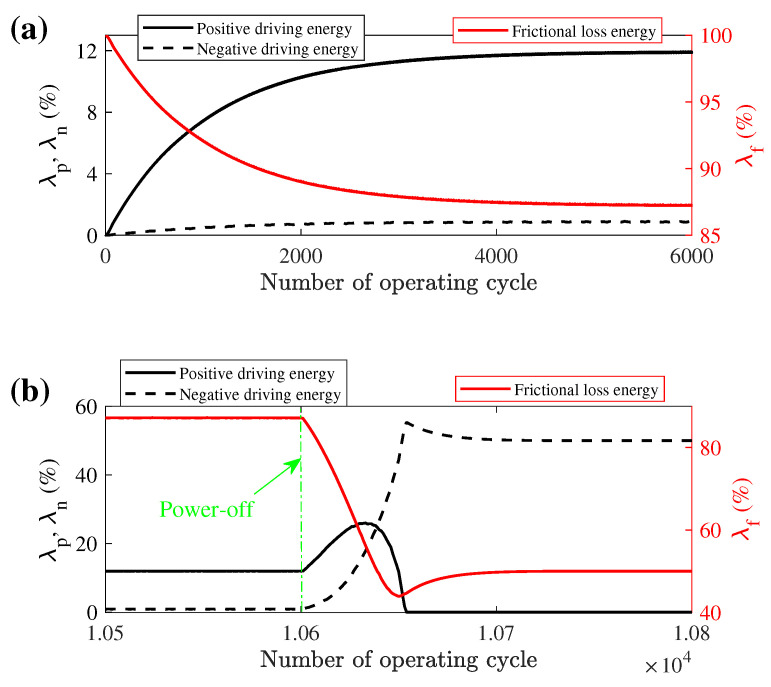
The transient energy transmission with the running cycle during: (**a**) ON transient state; (**b**) OFF transient state.

In this operating condition, the variation of the three energies transmission percentage with the number of running cycles during the ON and OFF transient states is depicted in Figure 27. In the ON transient state, the sliding friction of contact interfaces spends much energy which may be the reason for low efficiency in LUM, and the negative driving energy is a small minority because the frictional force is small when the stator and mover are in reverse motion. With the increase in the operating cycle, the proportion of frictional loss energy is decreased and the positive driving energy is increased. When the velocity of the mover reaches stable after about 2500 operating cycles, each of the energy percentages also reaches a balanced state. It can be found that the mover velocity has a significant effect on the three energy percentages even when the interfacial driving forces reach stability. After power-off, what is interesting is that the proportion of positive driving energy illustrates a short-term growth and then drops to zero. This can be explained as: (1) when the electric excitation is powered off, the rapid attenuation of the stator vibration leads to the velocity of the contact tip being close to the mover velocity first and farther away later, so the relative velocity between stator and mover will decrease first and then increase. (2) The positive driving energy is determined by the mover velocity and the amplitude of stator vibration; however, the frictional loss energy also relates to the velocity of the contact tip surface of the stator. The velocity of the contact tip decreases faster than the mover velocity. After the stator vibration stops, the negative driving energy also exists due to the mover still moving forward and it is equal to the frictional loss energy, and the stator acts as a brake. It is interesting that the percentage of the positive driving energy is almost the same change in velocity, so it can be used as a novel index to evaluate the performance ofthe motor.

### 5.3. Evaluation of the LUM’s Output Performance

The steady-state velocity of the mover is the most important index to evaluate the output performance of the LUM. The accurate evaluation of the output velocity of the mover under different input electric parameters would be useful to the design of the motor. In addition, the precision control of the motor depends heavily on the evaluation of the low-velocity state and the deadzone range of the motor, because the nonlinear stick–slip behavior between contact interfaces would occur and which could cause large position errors in the control of such motor [38]. For the LUM analyzed in this paper, the input parameters of the electric excitation signal of such a motor include the voltage amplitude, the voltage frequency, and the phase difference between the two excitation electrodes (CH1/CH2). Therefore, the variations of steady-state speeds of the mover with the amplitude, frequency, and phase difference of the excitation voltages are compared between the simulation and experiment at a fixed pre-pressure of 5 N. In addition, the steady-state driving trajectories of the contact tip under different output performances are also analyzed by numerical simulation for understanding the operational mechanism and guiding the design of LUM.

**Figure 28:**
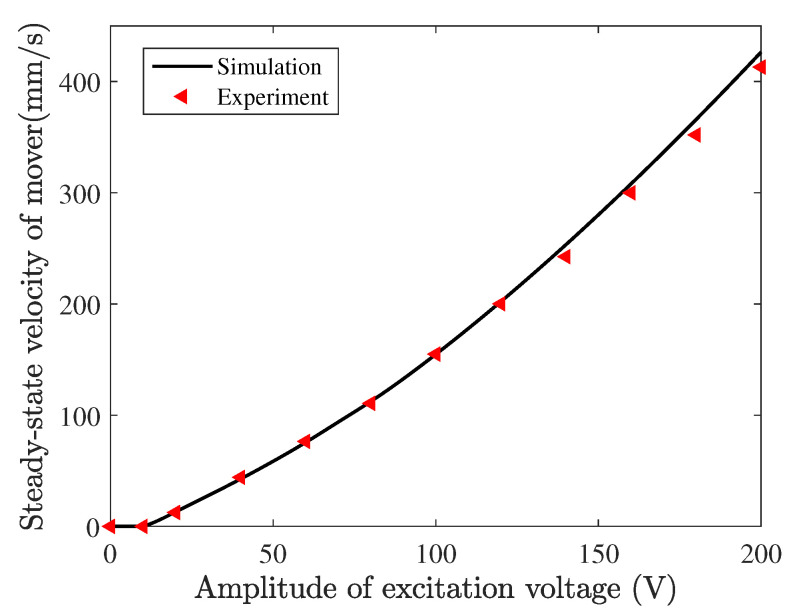
Velocity of the mover versus the amplitude of the excitation voltage.

**Figure 29:**
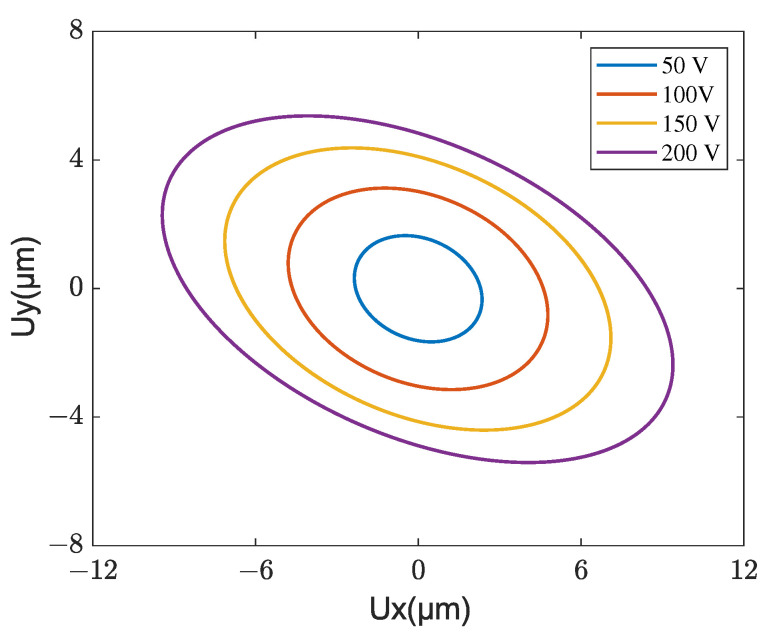
Driving trajectory of the contact tip for different voltage amplitudes.

Figure 28 shows the steady-state velocity of mover versus the amplitude of driving voltage obtained by experiment and simulation, where the frequency is fixed as 53 kHz and the phase difference between the two driving voltages is fixed as 90°. Good agreement between the simulation and experimental results is observed. It is worth noting that this simulated model can characterize the voltage amplitude deadzone (from 0 to 10 V) in which the stator can not drive the mover. This range of voltage amplitude is important for designing a compensating voltage in the amplitude modulation control of the motor. When the voltage amplitude is larger than 10 V, the velocity is approximately proportional to the voltage amplitude. In this case, the steady-state driving trajectories of the central position at the contact tip for several voltage amplitudes are shown in Figure 29 in which all trajectories are clockwise directions. It is found that the voltage amplitude only affects the size of the elliptical trajectory, and a bigger elliptical trajectory would increase the output performance of the motor.

**Figure 30:**
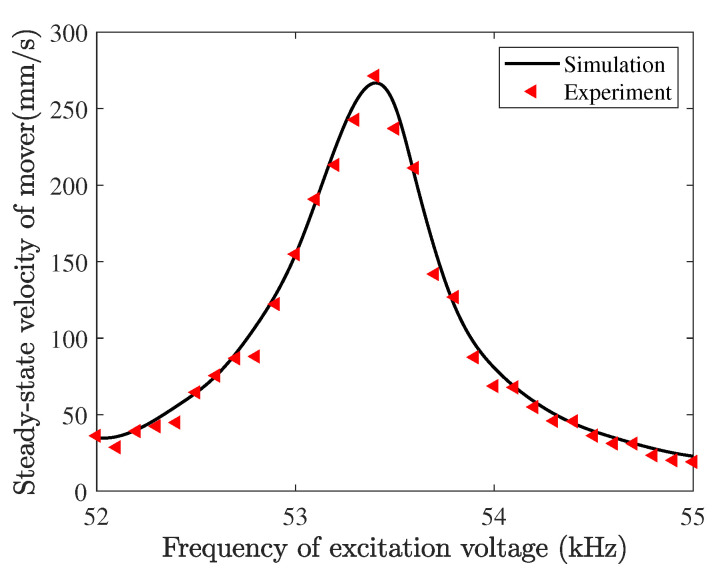
Velocity of the mover versus the frequency of the excitation voltage.

**Figure 31:**
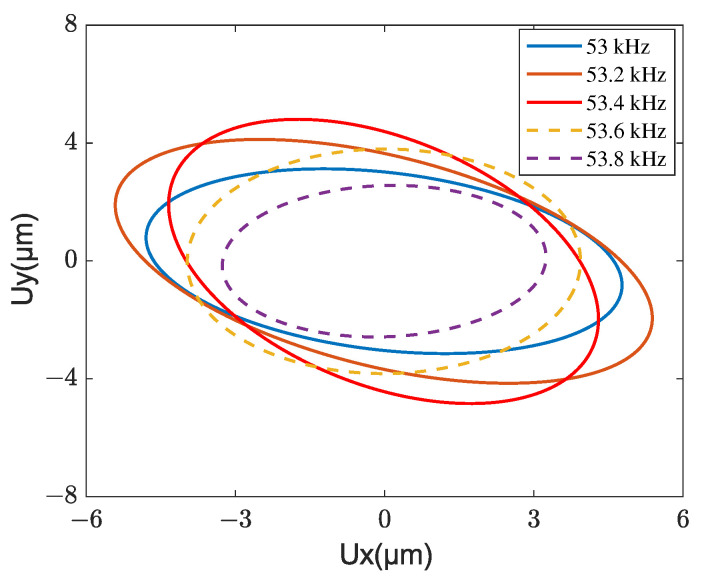
Driving trajectory of the contact tip for different voltage frequencies.

The steady-state velocities of a mover for different frequencies of driving voltage are tested and simulated, as shown in Figure 30. The voltage amplitude and the phase difference are fixed as 100 V and 90°, respectively. Within the frequency range from 52 to 55 kHz, the mover velocity rises first and starts to decline after passing 53.4 kHz which is called the motor reasonable frequency. The motor resonant frequency is higher than the designed stator resonant frequencies (53.3 kHz), which can be attributed to the effect of the stator–mover contact behaviors. The driving trajectories of the contact tip for several excitation frequencies near the motor resonant frequency are given in Figure 31. The solid and dashed lines denote the frequency below and above the stator resonant frequency, respectively. Additionally, the trajectories are clockwise directions. The excitation frequency not only affects the size of the elliptical trajectory but also the response phase difference between tangential and normal directions because the obliqueness of the elliptical trajectory is changed with different excitation frequencies. This is because the phase difference between excitation and response changes dramatically when the excitation frequency is close to the resonant frequency of the stator; however, the tangential and normal responses of the contact tip mainly rely on two resonant modes of the stator. It should be noted that the elliptical trajectory with the best performance has the maximum normal amplitude and obliqueness, however, the tangential amplitude is not maximum. This indicated that the motor performance can also be improved by adjusting the obliqueness of the elliptical trajectory in addition to increasing the vibration amplitude of the contact tip.

**Figure 32:**
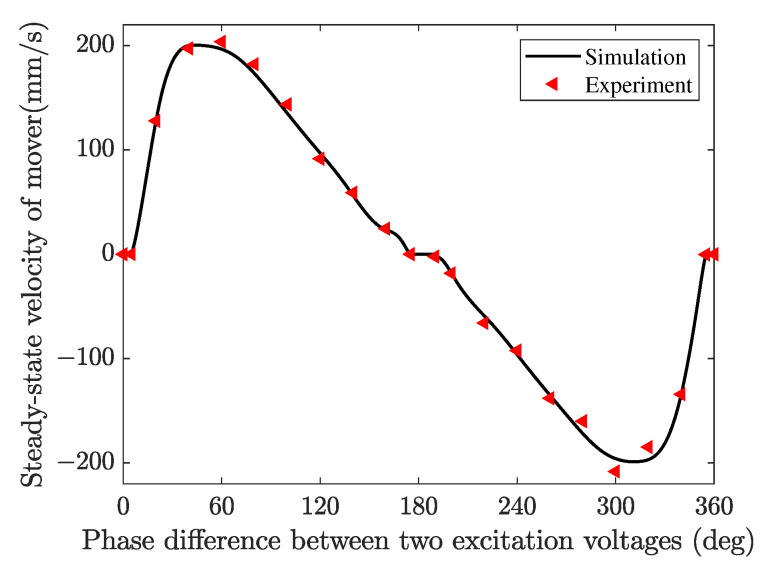
Velocity of the mover versus the phase difference between two excitation voltages.

**Figure 33:**
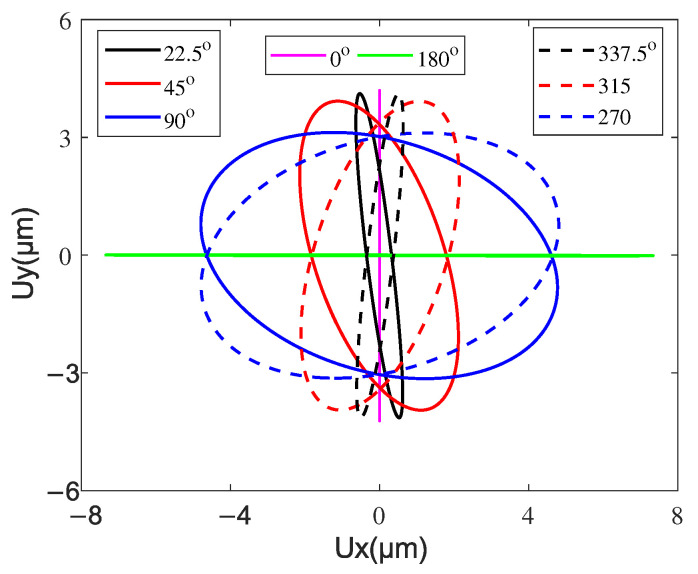
Driving trajectory of the contact tip for different excitation phase differences.

The phase difference between the two excitation electrodes (CH1/CH2) is an important parameter affecting the obliqueness of the elliptical trajectory, so it was chosen as the variable, and the steady-state velocity of the mover is measured and simulated, as shown in Figure 32. The amplitude and frequency of excitation voltage are fixed as 100 V and 53 kHz, respectively. The relationship between the velocity and phase closely resembles a sine wave shape. It can be found that the velocity of the mover reverses when the phase difference exceeded 180° due to the reverse driving of the contact tip. The maximum velocities in the forward and backward directions were observed around 45° and 315°. It is noted that three voltage phase deadzone (from 0° to 5°, from 175° to 185°, and from 355° to 360°) are recognized. Figure 33 shows several driving trajectories with different excitation phase differences. The solid and dashed lines denote clockwise and counterclockwise driving. When the phase difference is around 0°, the contact tip almost only generates normal displacement and the tangential displacement is too small to drive the mover. With the phase difference increasing from 0° to 175°, the tangential amplitude of the elliptic trajectory increases and the normal amplitude decreases, and the obliquity going from 90° to 0°. When the phase difference is around 180°, the contact tip mainly moves back and forth in the tangential direction. The small normal displacement results in the contact forces for back-and-forth driving being almost equal, i.e., the positive and negative driving effects of the contact tip almost cancel each other out in a running cycle so that the mover can not be driven. From 185° to 355°, the tangential amplitude of the elliptic trajectory decrease and the normal amplitude increase, and the obliquity goes from 0° to 90°. It can be concluded that the obliquity of the elliptic driving trajectory of the contact tip can be controlled by excitation phase modulation, and the obliquity of the elliptic trajectory has an optimal value for the interfacial energy transmission and output performance of the motor.

### 5.4. Effect of Micro-Topography Parameters on the Interfacial Force Transmission

The micro-topography of the contact interface has always been the top priority for a high-performance and low-wearing ultrasonic motor [39,40] because the motor is driven by the stator vibration in micro-scale. It is difficult to study the effect of micro-topography parameters on the interfacial forces transmission and the output performance of such motor directly because these parameters are interrelated with each other. Thus, simulations with only one variational parameter are conducted in this section, where the excitation voltage and the pre-pressure are respectively 100 V and 5 N, and other parameters in Table 1 are used. Effects of the roughness (σ), the areal density of asperity (η), the elastic constant ($E^{*}$), and the curvature radius of asperity (*R*) of the contact pairs on the interfacial forces transmission during steady state and the output velocity of mover are discussed, respectively.

**Figure 34:**
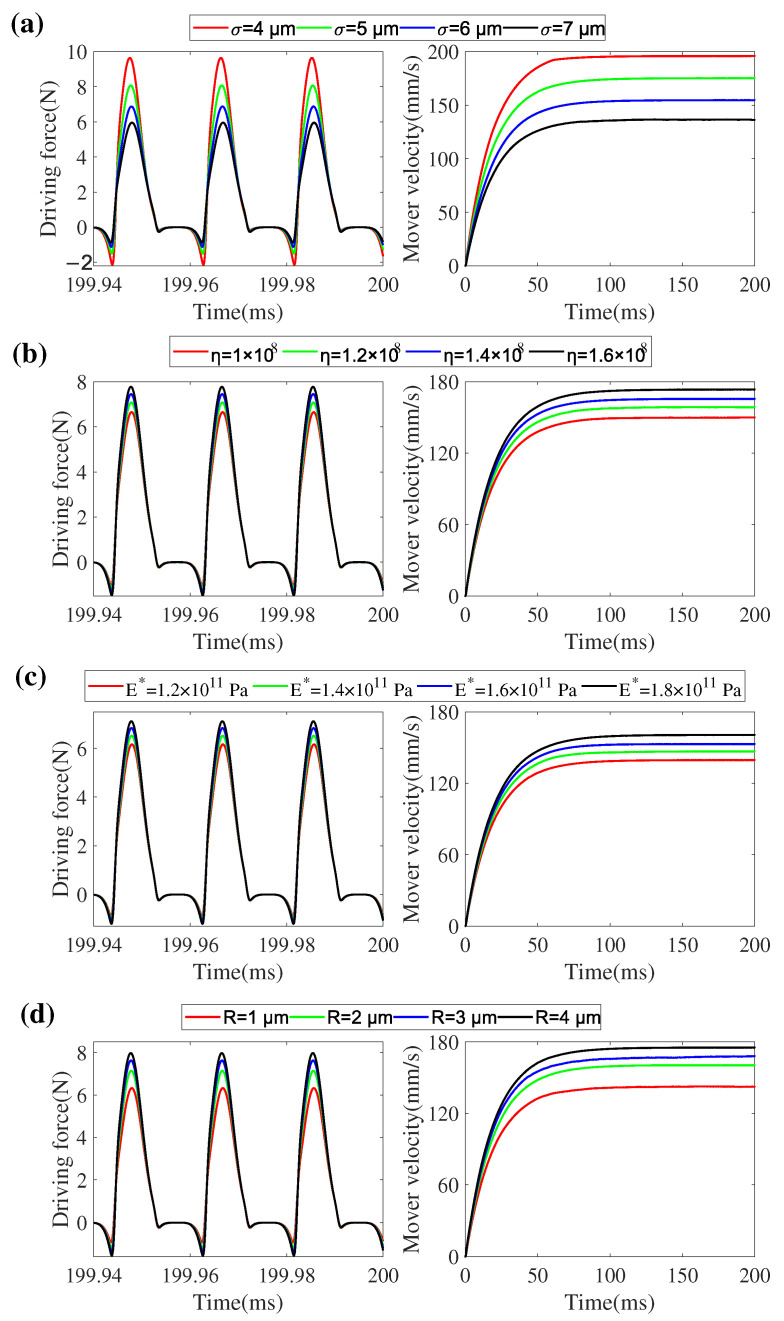
Effects of the micro-topography parameters on the driving force and the mover velocity response: (**a**) effect of roughness (σ), (**b**) effect of areal density of asperity (η), (**c**) effect of elastic constant ($E^{*}$), and (**d**) effect of the curvature radius of asperity (*R*).

Figure 34a shows the simulation results with different roughness, in which the tangential driving force and the mover velocity are given. With the increase of the roughness, both the output velocity of the mover and the driving force of the contact tip decreases. Similarly, the effect of areal density of asperity, elastic constant, and curvature radius of asperity are illustrated in Figure 34b–d, respectively. It can be seen that high areal density of asperity, elastic constant and curvature radius of asperity are beneficial to the transferred load of interface and then improve the performance of the motor. Although these micro-topography parameters do not change independently, these conclusions provide a theoretical basis for the choice of contact material and the manufacture of the contact surface, which would be helpful for improving the performance of LUMs.

## 6. Conclusions

A multi-point contact model considering a rough surface for the contact analysis between the stator and mover in an LUM is presented in this paper. Differing from the previous contact models for LUMs, this contact model not only considers the stator–mover contact as surface contact but also considers the interfacial micro-topography parameters. Furthermore, a piezoelectric bimorph sensor, which can simultaneously and quantitatively measure the dynamic normal and tangential contact forces between stator and mover, is introduced. Both the interfacial contact forces and the output performance of the sample motor are compared between the experiment and simulation. Results show that the model is effective for analyzing the static/dynamic contact mechanism and predicting the output performance. Based on this modeling method, the transient forces and energies transmission at the contact tip surface, the output performance of the motor under different electric excitation, and the effect of the interfacial micro-topography parameters on the interfacial force transmission and the output velocity of mover were simulated and discussed. Results show that stator vibration reaches stability much faster than the mover velocity which means that the response speed of the motor may be able to be improved. The carried forces at different positions of the contact tip surface not only exist hysteresis in time but different in amplitude which is important for investigating the wear of the contact interface. The interfacial energy transmission is defined and calculated in terms of the operating period, in which the percentage of positive driving can be used as a novel index to evaluate the motor’s performance. Both the size and obliquity of the elliptic driving trajectory of the contact tip influence the output performance, and the obliquity of the elliptic trajectory has an optimal value for interfacial energy transmission and output performance of the motor. The contact pair with a high areal density of asperity, elastic constant, curvature radius of asperity, and low roughness would be helpful for improving the force transmission at the contact interface and output performance of the mover.

Absolutely, other linear ultrasonic motors with different structures of the stator are also able to be simulated by the presented methodology because the stator is modeled based on the finite element method. Both the frictional contact behaviors and the motor performance are allowed to be evaluated in the simulation results, which is useful for designing a new type of LUM. Obtaining the carried force at different positions at the contact tip surface may be able to be employed to better analyze the wear of the contact pair, which will be investigated in future work.

## Figures and Tables

**Table 1:** Calibration coefficients of the sensor by experiment.

*h* (cm)	5	10	15	20	30
$c_{n}$ (N/V)	44.34	44.02	43.81	43.61	43.24
$c_{t}$ (N/V)	0.201	0.205	0.208	0.212	0.209

**Table 2:** Summary of model parameters.

Parameter	Description	Value	Units
Elastic constant for contact tip surface	$E_{1}$	$2.27\times 10^{11}$	Pa
Elastic constant for mover surface	$E_{2}$	$4\times 10^{11}$	Pa
Poisson’s ratio for contact tip surface	$\nu_{1}$	0.28	-
Poisson’s ratio for mover surface	$\nu_{2}$	0.41	-
Roughness of mover surface	$\sigma_{1}$	$2.7\times 10^{-6}$	m
Roughness of contact tip surface	$\sigma_{2}$	$5.4\times 10^{-6}$	m
Curvature radius of asperity	*R*	1.6 $\times 10^{-6}$	m
Areal density of asperity	η	$1.1\times 10^{8}$	$m^{-2}$
Stator–mover friction coefficient	μ	0.4	-
Stick–slip characteristic velocity	$\tilde{v}$	$1\times 10^{-6}$	m/s
Convergence tolerance	ϵ	$1\times 10^{-6}$	-
Time step of dynamic algorithm	δt	$1\times 10^{-7}$	s
Mass of mover	*m*	0.2	kg
Modal damping ratio	ξ	$5.5\times 10^{-3}$	
Damping coefficient of mover	*c*	1	N/(m/s)
Stiffness of preload spring	-	8	N/mm
Work frequency	-	53	kHz

**Figure A1:**
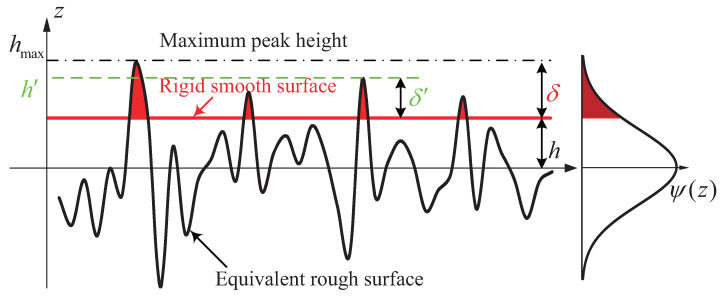
Normal contact of two stochastic rough surfaces.

## Appendix A. Equivalent Contact of Two Rough Surfaces

This appendix contains an equivalent approach to mathematically describe the normal contact between two rough surfaces presented in [24]. The contact of two elastic surfaces can be converted into the contact of a rigid smooth surface and an equivalent elastic rough surface, as shown in Figure A1.

The roughness σ of the equivalent rough surface is calculated by

(A1) $$\sigma =\sqrt{\sigma_{1}^{2}+\sigma_{2}^{2}},$$

where $\sigma_{1}$ and $\sigma_{2}$ are the roughness of two contact surfaces, and the equivalent elastic modulus $E^{*}$ satisfies

(A2) $$\frac{1}{E^{*}}=\frac{1-\nu_{1}^{2}}{E_{1}}+\frac{1-\nu_{2}^{2}}{E_{2}},$$

here *E* and ν are the elastic modulus and Poisson’s ratio, respectively. The subscripts ‘1’ and ‘2’ denote the two contact materials. The height distribution of the equivalent rough surface is expressed as

(A3) $$\psi \left(z\right)=\sqrt{2\pi \sigma }e^{-\frac{z^{2}}{2\sigma^{2}}}.$$
